# Homoleptic and heteroleptic ketodiiminate zinc complexes for the ROP of cyclic l-lactide†

**DOI:** 10.1039/d3ra06529d

**Published:** 2023-10-12

**Authors:** Eduard Glöckler, Leon Kapp, Christoph Wölper, Marcel Schumacher, André H. Gröschel, Stephan Schulz

**Affiliations:** a Faculty of Chemistry, University of Duisburg-Essen Universitätsstraße 7 45141 Essen Germany stephan.schulz@uni-due.de; b Faculty of Chemistry, University of Münster and Center for Soft Nanoscience (SoN) Busso-Peus-Strasse 10 48149 Münster Germany; c Center for Nanointegration Duisburg-Essen (CENIDE) Carl-Benz-Straße 199 47057 Duisburg Germany

## Abstract

Homo- and heteroleptic ketodiiminate zinc complexes L^1^_2_Zn_2_ (1, L^1^ = [Me_2_NC_2_H_4_NC(Me)CH]_2_CO), L^2^(ZnCp)_2_ (2, L^2^ = [Me_2_NC_3_H_6_NC(Me)CH]_2_CO, Cp = C_5_H_5_) and L^2^HZnCp* (3, Cp* = C_5_Me_5_) were synthesized and characterized by ^1^H and ^13^C NMR and IR spectroscopy as well as by elemental analysis and single crystal X-ray diffraction (sc-XRD, 2, 3). The catalytical activity of heteroleptic complexes 2 and 3 were tested in the ring-opening polymerization (ROP) of l-lactide. Homobimetallic complex 2 showed the highest activity and selectivity for the synthesis of cyclic polylactide (cPLLA; TOF = 17 460 h^−1^) at 100 °C in toluene solution, while linear polymers are formed with mononuclear complex 3.

## Abstract 2

We report the structures and activity of homo- and heteroleptic ketodiiminate zinc complexes in the ring-opening polymerization of l-lactide. Homobimetallic complex 2 selectively gave cyclic polylactide, while linear polymers were formed with mononuclear complex 3.

## Graphical abstract

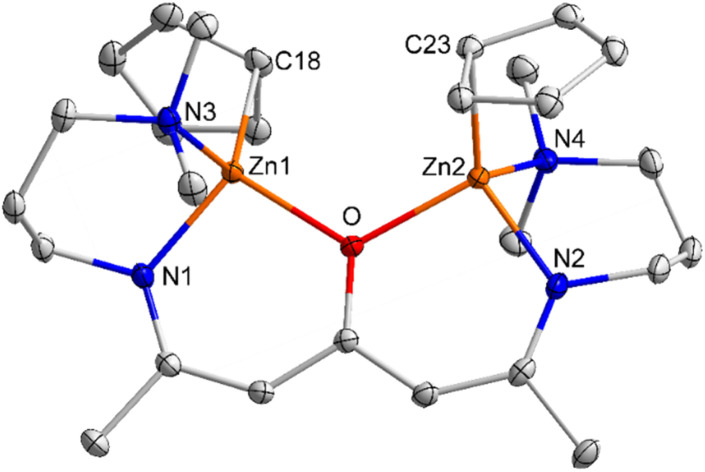

## Introduction

The use of polymers derived from renewable resources is considered an environmentally friendly and sustainable alternative to the use of petroleum-based plastics.^1–3^ In recent years, the physicochemical properties, biodegradability, and biocompatibility of aliphatic polyester have been extensively studied.^4–9^ Polylactide (PLA) is one of the most important polymers used in agriculture, packaging, pharmaceutical and biomedical fields. The physicochemical properties of such kind of polyesters can be modified by their molar mass, stereochemistry, typological structure and polymer architecture.^4,10,11^ Cyclic polyesters such as cyclic polylactide (cPLA) are also of interest since their properties differ from their linear counterparts in terms of melting temperature (*T*_m_), glass transition temperature (*T*_g_), viscosity, hydrodynamic volume, thermostability and morphology, respectively.^12–18^ However, unlike their linear and branched counterparts, the synthesis of cPLA with high molar mass continues to be a difficult scientific problem.^19,20^

Ring-closure and ring-expansion reactions are typically used for the synthesis of cyclic polymers.^21,22^ Unfortunately, ring-closure reactions often gave impure cyclic polymers with relatively low molar masses resulting from side-formation of linear polymers.^12^ In contrast, ring extension reactions often created cyclic polymers with higher molecular weights.^13^ One of the most effective ring-expansion catalysts for the synthesis of cyclic polylactide are N-heterocyclic carbenes (NHCs).^14–17^ Mechanistic analyses of the synthesis of cPLA revealed that neutral carbenes start the polymerization process by entering a zwitterionic state. The monomer is cyclized through transesterification, known as zwitterionic ring-opening polymerization (zROP). Carbene-catalysed reactions typically have a limited capacity for chain growth and, consequently, the production of high molecular weight polymers.^14–17^ In contrast, the addition of alcohols as initiators produced linear polylactides with higher molecular masses.^23–25^ In 2021, Arnold *et al.* reported a highly active and highly selective homogeneous Ce(iii)-NHC catalyst for the synthesis of high average molecular weight cyclic polylactides with turn-over-frequencies (TOFs) up to 864 000 h^−1^. This catalyst efficiently produces cyclic PLA from *rac*/l-lactide and other aliphatic cyclic polyesters, *i.e.*, *ε*-caprolactone or *β*-butyrolactone. The high activity results from synergistic effects between the Lewis acidic Ce(iii) centre and the N-heterocyclic carbene.^26^

In the last decades, main group metals such as alkaline or earth alkaline metals^27–29^ as well as zinc have been intensively studied as catalysts for the ROP of lactides and lactones due to their high activity, low toxicity and biocompatibility.^30–41^ In marked contrast, these type of metal complexes are rarely known for the preparation of cyclic polylactides. Recently, our group reported the synthesis of binuclear magnesium complexes and their capability for the synthesis of cPLA and cPCL with high TOF values up to 712 800 h^−1^ under mild reaction conditions.^42^ We herein report the synthesis of mono- and homodinuclear zinc ketodiiminate complexes and their catalytic activity for the synthesis of cPLA.

## Results and discussion

### Synthesis and characterization

**Scheme 1:**
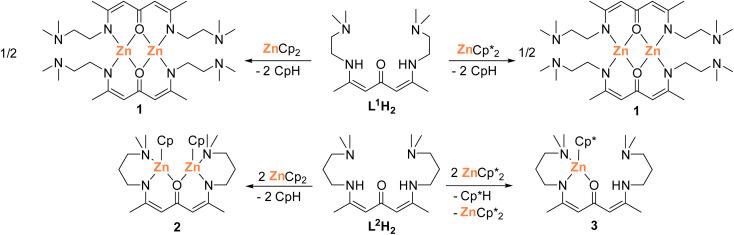
Synthesis of dinuclear homoleptic and mononuclear heteroleptic ketodiiminate zinc complexes 1–3.

Reactions of ketodiimine L^1^H_2_ with two equivalents of either Cp_2_Zn or Cp*_2_Zn (Cp = C_5_H_5_, Cp* = C_5_Me_5_) in toluene at 25 °C gave the homoleptic complex 1 (Scheme 1). *In situ*^1^H-NMR spectra of the reactions showed the disappearance of the N*H*-protons at 9.99 ppm and simultaneous formation of CpH (2.69, 6.08–6.50 ppm) and Cp*H (0.98–1.00, 1.74–1.80, 3.56–3.58 ppm), respectively (Fig. S7 and S8†). In contrast, reactions of ketodiimine L^2^H_2_ with two equivalents of Cp_2_Zn and Cp*_2_Zn in toluene at 25 °C gave the homobinuclear complex 2 and the mononuclear complex 3. Complex 3 was also obtained from the reaction of L^2^H_2_ with one equivalent of ZnCp*_2_, while reactions of L^1^H_2_ and L^2^H_2_ with one equivalent of ZnCp_2_ as well as of L^1^H_2_ with one equivalent of ZnCp*_2_ gave no pure compounds.

Complexes 2 and 3 were purified by recrystallization from solutions in toluene at −30 °C. The ^1^H NMR spectra of 2 (Fig. S11†) shows resonance due to the doubly deprotonated ligand L^2^ (4.25 ppm) and the Cp groups (6.32 ppm). All resonances are shifted to higher field compared to those of L^2^H_2_. In addition, broad triplet and multiplet signals indicate hindered rotations. In contrast, the ^1^H NMR spectrum of complex 3 (Fig. S14†) is more complex due to the asymmetric nature of the mono-deprotonated ligand L^2^H. The Cp* unit shows only one signal (1.89 ppm) caused by the fluctuation of the Cp* ring.

**Fig. 1:**
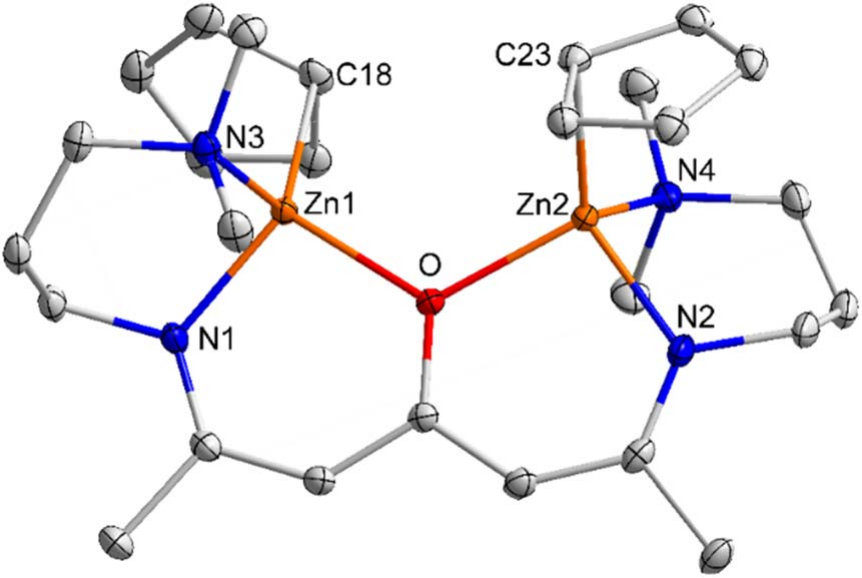
ORTEP representation of solid-state structure of complex 2. H atoms have been omitted for clarity and thermal ellipsoids are shown with 50% probability level. Selected bond lengths [Å] and angles [°]: Zn(1)–O 2.0266(8), Zn(1)–N(1) 1.9403(12), Zn(1)–N(3) 2.1311(13), Zn(1)–C(18) 2.0950(13), Zn(2)–O 2.0266(9), Zn(2)–N(2) 1.9437(11), Zn(2)–N(4) 2.1582(11), Zn(2)–C(23) 2.1051(12), N(1)–Zn(1)–O 99.59(4), N(1)–Zn(1)–C(18) 128.71(5), O–Zn(1)–C(18) 111.80(4), N(1)–Zn(1)–N(3) 92.25(4), O–Zn(1)–N(3) 110.57(4), C(18)–Zn(1)–N(3) 111.80(5), N(2)–Zn(2)–O 98.30(4), N(2)–Zn(2)–C(23) 126.99(5), O(1)–Zn(2)–C(23) 114.01(4), N(2)–Zn(2)–N(4) 92.63(5), O–Zn(2)–N(4) 111.71(4), C(23)–Zn(2)–N(4) 111.01(5).

**Fig. 2:**
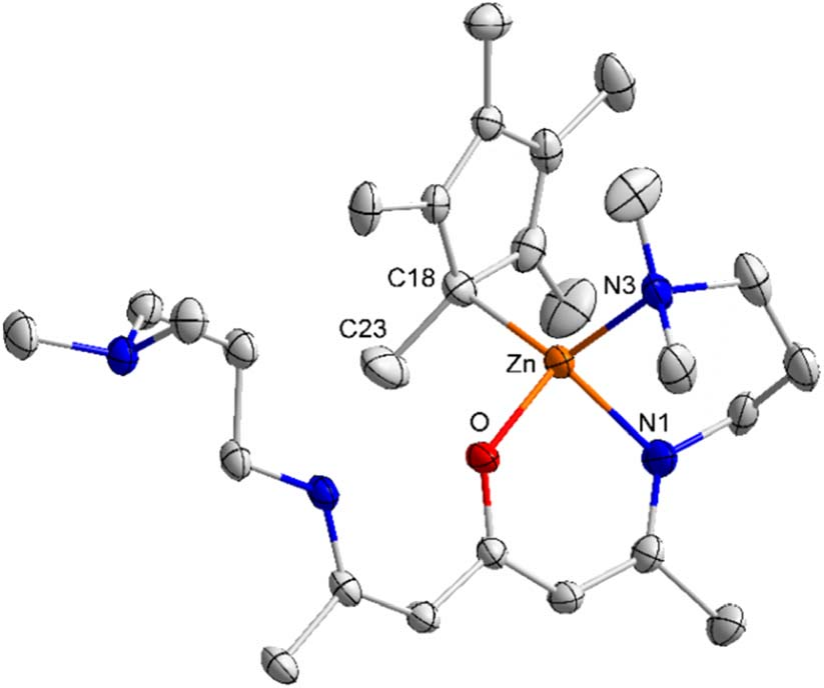
ORTEP representation of the solid-state structure of 3. H atoms and the second orientation of the side arm are omitted for clarity and thermal ellipsoids are shown with 50% probability level. Selected bond lengths [Å] and angles [°]: Zn(1)–O(1) 1.9821(16), Zn(1)–N(1) 1.985(2), Zn(1)–C(18) 2.087(2), Zn(1)–N(3) 2.127(2), O(1)–Zn(1)–N(1) 96.37(8), O(1)–Zn(1)–C(18) 107.63(8), N(1)–Zn(1)–C(18) 123.83(10), O(1)–Zn(1)–N(3) 105.16(8), N(1)–Zn(1)–N(3) 94.34(9), C(18)–Zn(1)–N(3) 124.98(8), C(23)–C(18)–Zn(1) 103.59(18).

Complexes 2 and 3 crystallize in the triclinic space group *P*1 (Fig. 1 and 2). The Zn atoms are tetrahedrally coordinated by the N, N, O heteroatoms of the ligand L^1/2^ and one η^1^-bonded Cp/Cp* group. The Zn–O bond lengths in complexes 2 (2.0266(8) Å) and 3 (1.9821(16) Å) as well as the Zn–N bond lengths in 2 (Zn(1)–N(1) 1.9403(12) Å, Zn(1)–N(3) 2.1311(13) Å) and 3 (Zn(1)–N(1) 1.985(2) Å, Zn(1)–N(3) 2.127(2) Å) are elongated compared to the sum of the covalent radii.^43^ In addition, the sum of the N(1)–Zn(1)–O/N(2)–Zn(2)–O (99.59(4)°/98.30(4)°), N(1)–Zn(1)–N(3)/N(2)–Zn(2)–N(4) (92.25(4)°/92.63(5)°) and O–Zn(1)–N(3)/O–Zn(2)–N(4) (110.57(4)°/111.71(4)°) bond angles of 2 are slightly larger than the sum of the N(1)–Zn(1)–O (96.37(8)°), N(1)–Zn(1)–N(3) (94.34(9)°), O–Zn(1)–N(3) (105.16(5)°) of compound 3, most likely resulting from the bulky Cp* substituents in compound 3.

### Catalytic studies

Complexes 1–3 were tested in the ROP of l-lactide (l-LA) at 100 °C in toluene in the absence of any co-initiator. The catalytic activity of each complex was tested with a monomer-to-metal ratio of 200 : 1 up to 1000 : 1 (Table 1). The resulting polymers were analysed by ^1^H NMR spectroscopy, while the *M*_n_ values were determined by gel permeation chromatography (GPC). Complex 1 turned out to be catalytically inactive, whereas complex 2 showed a high activity, and more than 90% of the monomer was converted within minutes (Table 1, entries 1–6). The conversion of 200 equivalent of l-LA occurred in 1 min, corresponding to a TOF value of 10 800 h^−1^.

**Table 1:** Polymerization data of l-LA using catalysts 2 and 3 and with catalysts I–III for comparison^a^

Entry	Catalyst	[M]o/[Zn]o	Time [min]	Conversion^b^ [%]	*M*_n_(GPC)^c^ [kg mol^−1^]	*M*_n_(Theo)^d^ [kg mol^−1^]	*Đ* e	TOF^f^ [h^−1^]
1	2	200 : 1	1	90	22.3	25.9	1.52	10 800
2	2	400 : 1	1.5	93	37.2	53.61	1.88	14 880
3	2	600 : 1	2	97	55.3	83.9	1.59	17 460
4	2	800 : 1	3	90	51.5	103.8	1.47	14 400
5	2	1000 : 1	4	80	58.2	115.3	1.47	12 000
6	2	200 : 1 + 200	1 + 1	98	56.0	56.5	1.58	10 855
7	2	200 : 1 + 200 + 200	1 + 1 + 4	98	54.6	84.7	2.28	5880
8	3	200 : 1	6	99	<10	28.5	—	1980
9	3	400 : 1	14	99	<10	57.1	—	1697
10	3	600 : 1	300	85	11.7	73.5	1.02	102
11 (ref. 36)	I^g^	200 : 1	210	94	8.1	27.1	1.4	54
12 (ref. 37)	II^g^	200 : 1	35	93	27.2	26.8	1.2	318
13 (ref. 42)	III^g^	200 : 1	15	97	31.9	27.9	1.58	776

a Polymerization conditions: all polymerizations were performed 100 °C in toluene.

b Determined by ^1^H NMR spectroscopy.

c Measured by GPC at 40 °C in CHCl_3_, relative to poly(ethylene oxide) standards.

d *M* ^theo^_n_ at 100% conversion = [M]_0_/[Zn]_0_ × mol. wt of monomer.

e Measured by GPC.

f TOFs were calculated as (mol of monomer consumed)/(mol of catalyst × time of polymerization).

g At ambient temperature (25 °C) in toluene or CH_2_Cl_2_.

The resulting *M*_n_ of 22.3 kg mol^−1^ is close to the theoretically expected value (25.9 kg mol^−1^), and a moderate dispersity *Đ* of 1.52 was obtained. The activity of complex 2 is almost maintained when the monomer-to-metal ratio is increased up to 1000 : 1. However, the gap between the expected and obtained *M*_n_ values of the polymers increased with increasing dilution of the catalyst. Thus, only polymers with a maximum *M*_n_ of 58.2 kg mol^−1^ and moderate *Đ*s (1.47–1.88) were obtained. Moreover, complex 2 showed “living” polymerization character in the ROP of l-LA using a monomer-to-metal ratio of 200 : 1 in toluene at 100 °C. After full conversion of the initially added l-LA, a second fraction of 200 eq. of l-LA was added, whose turnover occurred after 70 seconds (Table 1, entry 6). The GPC data of the resulting polymer still showed a monomodal peak with a similar *Đ* compared to the 200 : 1 obtained polymer (Fig. S15†), while the *M*_n_ value of the resulting polymer was higher than that formed with only 200 eq. of l-LA. In marked contrast the addition of a third fraction of 200 eq. of l-LA, which was fully converted within 4 minutes, did not result in an increased *M*_n_ value of the resulting polymer (Table 1, entry 7). This indicates that the molecular weight of the resulting cyclic polymer is somehow restricted to >60 kg mol^−1^, which is supported by the broadened *Đ* value of 2.28.

We also investigated the catalytic activity of the mono-substituted zinc complex 3 in the ROP of l-LA. *In situ*^1^H NMR spectroscopy showed full conversion of the monomers after 6 minutes for a monomer-to-metal ratio of 200 : 1 and after 14 minutes for a monomer-to-metal ratio of 400 : 1. GPC analyses showed that the obtained polymers had a lower molar mass than 10 kg mol^−1^, which made it difficult to determine the molecular weight distribution qualitatively (Table 1, entries 8 and 9). Also, a tremendous decrease in the catalytic activity of complex 3 was observed at a monomer-to-metal ratio of 600 : 1, converting 85% of the monomer after 5 hours, yielding a *M*_n_ of 11.74 kg mol^−1^ with a narrow *Đ* of 1.02 (Table 1, entry 10).

**Scheme 2:**
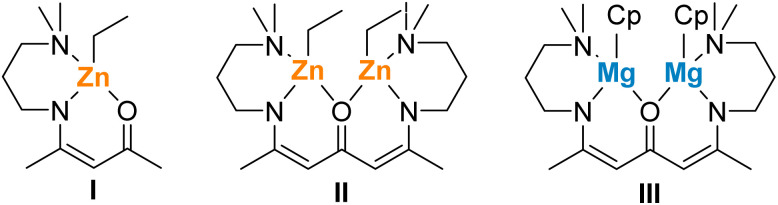
Catalytically active mono- and bimetalic catalysts I, II and III.^36,37,42^

We already noted the enhanced catalytic activity of homobimetallic complexes compared to similar monometallic β-ketoiminate zinc and ketodiiminate zinc complexes.^36,37,42^ The higher catalytic activity of complex 2 compared to complex 3 most likely results from the presence of the second metal centre, allowing to proceed the polymerization process at two active centres at the same time. Homo- and heterobimetallic catalysts are widely investigated in recent years due to the potential beneficial effect of two metals, which can affect the mechanism, and thus the activity, of the catalyst.^44,45^ Comparing complex 2 with complex II (Scheme 2), significant higher polymerization rates were observed with complex 2 (TOF = 10 800 h^−1^ (2); 318 h^−1^ (II)), while the formation of a polymer with higher molecular weight and lower molecular weight distribution was observed with catalyst II, indicating a less controlled polymerization process due to the bulkier Cp moieties. When comparing complex 2 with the similar Mg complex III, it is noticeable that complex III already showed a high catalytic activity (TOF = 776; Table 1, entry 13) under milder reaction conditions (25 °C). The resulting PLLA showed similar *M*_n_ and *Đ* values, but higher molecular weight PLLA was obtained with catalyst III and increasing monomer-to-metal ratio. These findings show that the catalytic activity is not only largely influenced by the metal centre (Zn *vs.* Mg) but also by the Cp* substituent. The introduction of a Cp*Zn moiety to ketodiiminate complexes yields lower molecular weight polymers.^42,46^

### Kinetic studies

**Fig. 3:**
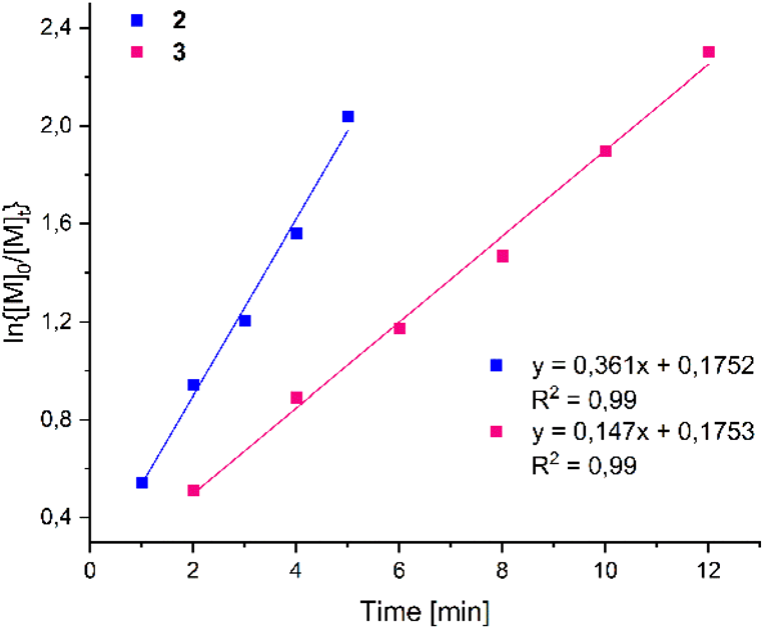
Semi-logarithmic plots of l-LA conversion at 100 °C initiated by complexes 2 and 3*versus* time: [l-LA]_0_/[Zn]_0_ = 200 in toluene.

Kinetic studies with complexes 2 and 3 were performed in toluene at 100 °C with monomer-to-metal ratios [LA]_0_/[Zn]_0_ of 200 : 1 in a *J*-Young NMR tube. The ln{[M]_0_/[M]_*t*_} ratio was calculated by integration of the Me proton peaks in the ^1^H NMR spectra from the polymer and the monomer (Fig. 3). We did not observe any induction period in the catalytic studies, and the plots of ln{[M]_0_/[M]_*t*_} *versus* time were for both complexes linear, showing a first-order dependency of the polymerization rates with respect to the monomer concentration. The apparent rate constants (*k*_app_) were calculated from the slope of the observed plots (3.61 × 10^−1^2, 1.47 × 10^−1^3), and the homobimetallic complex 2 was found to be more active than the mononuclear complex 3.

In addition to the GPC analysis, the resulting PLLA was also analysed by ^1^H NMR and IR spectroscopy as well as by matrix-assisted laser desorption ionization time-of-flight (MALDI-ToF) mass spectroscopy. Linear PLLA was formed with the mononuclear zinc complex 3 as was proven by the detection of a hydroxyl (OH) chain-end group using both MALDI-ToF mass and IR spectroscopy (Fig. S22 and S23†).

In marked contrast, the MALDI-ToF spectra of the low-molecular-weight PLLA ([l-LA] : [Cat] = 50 : 1) obtained with complex 2 showed a peak series, which are equally separated by *m*/*z* = 72 au, which indicates the formation of cyclic polymer (72*n* + 23 is assigned to *n*(C_3_H_4_O_2_) + Na^+^, Fig. S20†). The absence of the polymer chain end was proven by ^1^H NMR and IR spectroscopy (Fig. S19 and S21†), confirming the selective formation of cyclic polylactide (cPLLA). In marked contrast, the formation of linear PLLA was observed with the similar binuclear ketodiiminate zinc complex II, while the ketodiiminate Mg complex III also gave cPLA. These findings indicate a rather strong influence of the Cp ligand on the polymerization mechanism, since the heteroleptic binuclear zinc complexes 2 and II only differ by one substituent (Cp in 2*vs.* Et in II).

Since cyclic polymers were only formed with the bimetallic zinc complex 2 and previously reported magnesium complex III as well as its Cp*-substituted analogue, we assume the same polymerization mechanism as reported for the bimetallic ketodiiminate magnesium complexes.^42^ Both Zn atoms in complex 2 are initially coordinated by one lactide molecule. Nucleophilic attack of the Cp ligand at the electrophilic carbonyl unit of the coordinated lactide causes the ring opening followed by the formation of the catalytic active alkoxide species. The propagation process of the polymer performs through stepwise coordination of a lactide molecule to a zinc centre followed by insertion of an alkoxy group by breaking the Zn–O bond. The formation of the cyclic polymer occurs most likely by an intramolecular transesterification reaction, yielding a limited molecular weight polymer >60 kg mol^−1^ with moderate molecular weight distributions (Table 1, entries 1–7).^42^

## Experimental

### General experimental details

The reactions were performed under standard Schlenk and glovebox techniques under argon atmosphere, which was dried by passing through preheated Cu_2_O pellets and molecular sieves columns. Toluene and *n*-hexane were dried using a mBraun Solvent Purification System (SPS) and stored over molecular sieves (3 Å) under argon atmosphere. Deuterated solvents were dried over molecular sieves (3 Å). Karl Fischer titration of the dry solvents showed values below 2 ppm. Amines were commercially available (Sigma-Aldrich) and used as received. l-LA was sublimed three times and stored in a glovebox. ZnCp_2_ and ZnCp*_2_ were prepared according to literature methods.^47^ 2,4,6-heptatrione was prepared according to a slightly modified literature procedure.^48^

### Materials and methods

^1^H and ^13^C NMR spectra were measured at 297 K in CD_2_Cl_2_ using a Bruker Avance 300 spectrometer with a QNP probe head (^1^H: 300 MHz, ^13^C: 75 MHz) or Bruker Avance 500 (^1^H: 400 MHz, ^13^C: 125 MHz) and referenced to the solvent shifts (CD_2_Cl_2_: ^1^H = 5.32 ppm, ^13^C = 53.84 ppm). IR spectra were determined using an ALPHA-T FT-IR spectrometer equipped with a single reflection ATR sampling module in a glovebox. MALDI-ToF mass spectra were recorded with a Bruker UltrafleXtreme MALDI-ToF mass spectrometer (Bruker Daltonik). The polymers were dissolved in THF (10 mg mL^−1^) using dihydroxy benzoic acid as the matrix in THF (20 mg mL^−1^). The mass spectra of the polymer samples were received using the reflective positive ion mode. An externally calibration was done using poly(methylmethacrylate) standards. Microanalyses were observed with a PerkinElmer Series 11 analyzer by the Elementaranalyse Labor of the University of Duisburg-Essen. Melting points were determined in glass capillaries sealed with grease and are not corrected.

#### Gel permeation chromatography (GPC)

The number-average molecular weight (*M*_n_), molecular weight distribution (*M*_W_, MWD) and dispersity (*Đ* = *M*_w_/*M*_n_) of all obtained polymers were determined by GPC on a 1260 Infinity instrument (Polymer Standard Service, Mainz) equipped with 3 SDV columns (pore sizes 10^4^, 2 × 10^3^ Å) and a SECcurity differential refractometer. PLA and ε-CL was measured using HPLC grade CHCl_3_ as eluent at a flow rate of 1.0 mL min^−1^ at 40 °C (column oven TCC6000). *M*_n_ and *Đ* values of all polymers were determined relative to polyethylene oxide standards with narrow distribution and molecular weights ranging from 750 to 400 000 g mol^−1^ and evaluated with the WinGPC UniChrom software. For preparation, polymers were dissolved in CHCl_3_ (2 mg mL^−1^) and passed through a syringe filter with a pore size of 0.2 μm.

#### Synthesis of ligand L^1^H_2_ and L_2_H_2_

Two equivalents of *N*,*N*-dimethylethylenediamine or *N*,*N*-dimethylpropylenediamin (70.5 mmol) were added dropwise to a solution of one equivalent of 2,4,6-heptatrione (5 g, 35.17 mmol) in 50 mL of THF at ambient temperature (25 °C). The solution was stirred for 12 h, all volatiles evaporated under reduced pressure, and the resulting crude product was recrystallized twice from saturated solutions in *n*-hexane at 0 °C. L^1^H_2_ and L^2^H_2_ were isolated as orange crystals by filtration and dried under vacuum.

##### [L^1^H_2_]

Yield: 8.44 g (85%). Mp: 64 °C. Anal. calc. for C_15_H_30_N_4_O: C, 63.81; H, 10.71; N, 19.83; Found: C, 63.8; H, 10.8; N 20.3; ^1^H NMR (300 MHz, CD_2_Cl_2_, 300 K): *δ* = 1.86 (s, 6H, NCC*H*_3_), 2.24 (s, 12H, N(C*H*_3_)_2_), 2.41 (t, ^3^*J*_HH_ = 6.6 Hz, 4H, C*H*_2_N(CH_3_)_2_), 3.23–3.29 (m, 4H, CNC*H*_2_), 4.60 (s, 2H, OCC*H*), 9.99 (s, 2H, N*H*). ^13^C NMR (75.5 MHz, CD_2_Cl_2_, 300 K): *δ* = 19.6 (NC*C*H_3_), 41.5 (*C*H_2_N(CH_3_)_2_), 45.9 (CH_2_N(*C*H_3_)_2_), 60.2 (N*C*H_2_CH_2_), 95.8 (OC*C*H), 158.6 (*C*N), 190.2 (*C*O). ATR IR: *ν* = 2963, 2928, 2848, 2802, 2751, 1616, 1559, 1430, 1378, 1267, 1156, 1088, 1059, 1010, 857, 832, 786, 681, 620, 540 cm^−1^.

##### [L^2^H_2_]

Yield: 9.82 g (90%). Mp: 70 °C. Anal. calc. for C_17_H_34_N_4_O: C, 65.76; H, 11.04; N, 18.05. Found: C, 65.90; H, 11.26; N 18.35. ^1^H NMR (300 MHz, CD_2_Cl_2_, 300 K): *δ* = 1.62–1.71 (m, 4H, NCH_2_C*H*_2_), 1.86 (s, 6H, (NCC*H*_3_)_2_), 2.17 (s, 12H, N(C*H*_3_)_2_), 2.28 (t, ^3^*J*_HH_ = 6.9 Hz, 4H, C*H*_2_N(CH_3_)_2_), 3.18–3.24 (m, 4H, CNC*H*_2_), 4.59 (s, 2H, OCC*H*), 10.08 (s, 2H, N*H*). ^13^C NMR (75.5 MHz, CD_2_Cl_2_, 300 K): *δ* = 19.4 (NC*C*H_3_), 29.4 (NCH_2_*C*H_2_), 41.3 (*C*H_2_N(CH_3_)_2_), 45.8 (CH_2_N(*C*H_3_)_2_), 57.3 (N*C*H_2_CH_2_), 95.5 (OC*C*H), 159.2 (*C*N), 190.3 (*C*O). ATR IR: *ν* 2963, 2928, 2848, 2802, 2751, 1616, 1559, 1430, 1378, 1267, 1156, 1088, 1059, 1010, 857, 832, 786, 681, 620, 540 cm^−1^.

#### Synthesis of compound 1–3

Solutions of L^1^H_2_ or L^2^H_2_ in 5 mL of toluene were added dropwise at 25 °C to solutions of two equivalents of the cyclopentadienyl zinc complexes (Cp_2_Zn or Cp*_2_Zn) in 5 mL of toluene. The reaction mixtures were stirred for 3 h at ambient temperature. All volatiles were evaporated under reduced pressure and the resulting crude products were washed with 10 mL of *n*-hexane to give orange/red powders, which were recrystallized from saturated solutions in toluene at −30 °C. Complexes 2 and 3 were isolated by filtration and dried under vacuum.

##### L^1^_2_Zn_2_ (1)

Either ZnCp_2_ (277 mg, 1.42 mmol) or ZnCp*_2_(476 mg, 1.42 mmol), L^1^H_2_ (200 mg, 0.71 mmol). Yield: 155 mg (43%). Mp 210 °C (dec.). Anal. calc. for C_30_H_56_N_8_O_2_Zn_2_: C, 52.10; H, 8.16; N, 16.20. Found: C, 53.01; H, 8.02; N, 16.18. ^1^H NMR (400 MHz, C_6_D_6_, 300 K): *δ* = 1.89 (s, 12H, (NCC*H*_3_)_2_), 2.19 (s, 24H, N(C*H*_3_)_2_), 2.27–2.30 (br, 8H, C*H*_2_N(CH_3_)_2_), 3.09–3.15 (m, ^3^*J*_HH_ = 6 Hz, 4H, NCH_2_C*H*_2_), 3.28–3.37 (m, ^3^*J*_HH_ = 7 Hz, 4H, NCH_2_C*H*_2_), 4.82 (s, 4H, OCC*H*). ^13^C NMR (150 MHz, C_6_D_6_, 300 K): *δ* = 22.8 (NC*C*H_3_), 47.5 (CH_2_N(*C*H_3_)_2_), 52.1 (*C*H_2_N(CH_3_)_2_), 61.1 (N*C*H_2_CH_2_), 93.5 (OC*C*H), 166.8 (*C*N), 180.8 (*C*O). ATR IR: *ν* = 2950, 2908, 2855, 2844, 2716, 1626, 1533, 1464, 1411, 1398, 1326, 1287, 1268, 1253, 1241, 1204, 1179, 1096, 1080, 1020, 1003, 937, 860, 780, 728, 709, 644, 617, 556, 505, 463, 455, 443, 414 cm^−1^.

##### L^2^Zn_2_Cp_2_ (2)

ZnCp_2_ (200 mg, 1.02 mmol), L^2^H_2_ (160 mg, 0.51 mmol). Yield: 275 mg (95%). Mp. 140 °C (dec.). Anal. calc. for C_27_H_42_Zn_2_N_4_O: C, 56.95; H, 7.42; N, 9.86. Found: C, 54.93; H, 7.41; N, 9.89. ^1^H NMR (300 MHz, CD_2_Cl_2_, 300 K): *δ* = 1.39–1.43 (m, ^3^*J*_HH_ = 5.4 Hz, 4H, NCH_2_C*H*_2_), 1.74 (s, 6H, (NCC*H*_3_)_2_), 2.46 (s, 12H, N(C*H*_3_)_2_), 2.49 (br, 4H, C*H*_2_N(CH_3_)_2_), 3.05 (br, 2H, CNC*H*_2_), 4.23 (s, 2H, OCC*H*), 6.31 (s, 10H, C_5_*H*_5_). ^13^C NMR (75.5 MHz, CD_2_Cl_2_, 300 K): *δ* = 21.4 (NC*C*H_3_), 28.3 (NCH_2_*C*H_2_), 47.4 (CH_2_N(*C*H_3_)_2_), 49.8 (*C*H_2_N(CH_3_)_2_), 62.8 (N*C*H_2_CH_2_), 93.9 (OC*C*H), 109.7 (*C*_5_H_5_), 165.1 (*C*N), 176.2 (*C*O). ATR IR: *ν* = 3061, 2992, 2905, 2864, 2834, 1558, 1479, 1444, 1408, 1398, 1378, 1328, 1302, 1251, 1224, 1171, 1148, 1101, 1084, 1055, 1034, 1020, 998, 980, 906, 864, 837, 812, 781, 749, 720, 656, 617, 583, 556, 475, 443, 411 cm^−1^.

##### L^2^HZnCp* (3)

ZnCp*_2_ (200 mg, 0.6 mmol), L^2^H_2_ (93 mg, 0.3 mmol). Yield: 75 mg (49%). Mp 210 °C. Anal. calc. for C_27_H_48_ZnN_4_O: C, 63.58; H, 9.49; N 10.98. Found: C, 64.22; H, 9.86; N, 10.11. ^1^H NMR (300 MHz, CD_2_Cl_2_, 300 K): *δ* = 1.42–1.49 (m, ^3^*J*_HH_ = 5.4 Hz, 2H, NCH_2_C*H*_2_), 1.65–1.73 (m, ^3^*J*_HH_ = 6 Hz, 2H, NCH_2_C*H*_2_), 1.82 (s, 3H, CNC*H*_3_), 1.87 (s, 3H, CNC*H*_3_), 1.89 (s, 15H, C_5_(C*H*_3_)_5_), 2.07 (s, 6H, N(C*H*_3_)_2_), 2.18 (s, 6H, N(C*H*_3_)_2_), 2.22–2.26 (t, ^3^*J*_HH_ = 6 Hz, 2H, C*H*_2_N(CH_3_)_2_), 2.29–2.34 (t, ^3^*J*_HH_ = 7.5 Hz, 2H, C*H*_2_N(CH_3_)_2_), 3.18–3.25 (q, 2H, ^3^*J*_HH_ = 7 Hz, CNC*H*_2_), 3.28–3.31 (q, 2H, ^3^*J*_HH_ = 4.5 Hz, CNC*H*_2_), 4.34 (s, 1H, OCC*H*), 4.44 (s, 1H, OCC*H*). ^13^C NMR (75.5 MHz, CD_2_Cl_2_, 300 K): *δ* = 12.3 (C_5_(*C*H_3_)_5_), 19.9 (NC*C*H_3_), 20.9 (NC*C*H_3_), 27.9 (NCH_2_*C*H_2_), 30.0 (NCH_2_*C*H_2_), 41.8 (N*C*H_2_CH_2_), 45.9 (CH_2_N(*C*H_3_)_2_), 47.6 (CH_2_N(*C*H_3_)_2_), 49.3 (N*C*H_2_CH_2_), 57.7 (*C*H_2_N(CH_3_)_2_), 62.0 (*C*H_2_N(CH_3_)_2_), 95.1 (OC*C*H), 96.0 (OC*C*H), 113.7 (*C*_5_(CH_3_)_5_), 155.1 (*C*N), 168.7 (*C*N), 182.2 (*C*O). ATR IR: *ν* = 3286, 2944, 2929, 2903, 2843, 2810, 2785, 2758, 1613, 1546, 1498, 1456, 1436, 1400, 1329, 1306, 1282, 1259, 1228, 1195, 1151, 1098, 1060, 1007, 971, 942, 896, 861, 837, 783, 762, 720, 645, 577, 477, 442 cm^−1^.

#### General procedure for the polymerization of l-LA

As an example, 0.5 g of lactide were dissolved in 4 mL of toluene in a 25 mL Schlenk tube and solutions of 17.34 μmol of complexes 2 or 3 in 1 mL of toluene were added. The solutions were stirred at the ambient temperature (25 °C) until the polymerizations were complete. To determine the conversion of the lactide by ^1^H NMR analysis, 1 mL of the reaction mixture was collected and dried by reduced pressure. To quench the reactions, the mixture was transferred into 20 mL of *n*-hexane, and the resulting polymers were filtered and dried for 12 h in an oven at 40 °C. The same procedure was used for higher monomer-to-metal ratios. All polymerization reactions were performed twice.

#### Single crystal X-ray analysis

The crystals were mounted on nylon loops in inert oil. Data were collected on a Bruker AXS D8 Kappa diffractometer with APEX2 detector (monochromated Mo Kα radiation, *λ* = 0.71073 Å) at 100(2) K. The structures were solved by Direct Methods (SHELXS-2013)^49^ and refined anisotropically by full-matrix least-squares on *F*^2^ (SHELXL-2017).^50,51^ Absorption corrections were performed semi-empirically from equivalent reflections on basis of multi-scans (Bruker AXS APEX3). Hydrogen atoms were refined using a riding model or rigid methyl groups. The crystal of complex 3 was a non-merohedral twin of two components. The model was refined against HKLF5 data. The coordinated side arm is disordered over two positions. Its corresponding bond lengths and angles were restrained to be equal (SADI) and RIGU restraints were applied to the anisotropic displacement parameters. For C8, C8′, C12, and C12′ additional SIMU restraints were used.

## Conclusions

Ketodiiminate Zn complexes 1–3 were synthesized and the catalytic activity of the dinuclear (2) and mononuclear (3) heteroleptic complexes in the ROP of l-lactide studied. The highest catalytic activity was observed with complex 2, which selectively gave cyclic polylactide (cPLA) with low molecular weight >60 kg mol^−1^ and moderate *Đ* (1.47–1.88). In contrast, linear PLLA was obtained with complex 3. The polymerization reactions with complexes 2 and 3 is expected to proceed *via* coordination insertion mechanism (CIM), whereas cPLA is formed by cyclization of the polymer chains through intramolecular transesterification in case of complex 2.

## Author contributions

E. G. and L. K. performed the experiments including catalytic studies, C. W. the single crystal X-ray diffraction, and M. S. the GPC experiments. The work was supervised by A. H. G. and St. S. The manuscript was written through contributions of all authors. All authors have given approval to the final version of the manuscript.

## Conflicts of interest

There are no conflicts to declare.

## Supplementary Material

RA-013-D3RA06529D-s001

RA-013-D3RA06529D-s002
