# Supplementary material for: Homoleptic and heteroleptic ketodiiminate zinc complexes for the ROP of cyclic l-lactide
Source: RSC Adv. 2023 Oct 12;13(43):29879–85. doi: 10.1039/d3ra06529d (PMC10568404; doi:10.1039/d3ra06529d)
Supplement: RA-013-D3RA06529D-s001 [file RA-013-D3RA06529D-s001.pdf]

Electronic Supplementary Material (ESI) for RSC Advances.
This journal is © The Royal Society of Chemistry 2023

Supporting Information

Homoleptic and heteroleptic ketodiiminate zinc complexes for the ROP of cyclic *L*-Lactide

*Eduard Glöckler, Leon Kapp, Christoph Wölper, Marcel Schumacher, André H. Gröschel, Stephan Schulz**

Content

I. Spectroscopic Characterization

Figures S1-S3. ^{1}H, ^{13}C NMR, and IR spectra of L^1H_2.

Figures S4-S6. ^{1}H, ^{13}C NMR and IR spectra of L^2H_2.

Figure S7. ^{1}H NMR spectrum of the reaction of L^1H_2 with 2 eq. of $ZnCp^*_2$.

Figure S8. ^{1}H NMR spectrum of the reaction of L^1H_2 with 2 eq. of $ZnCp_2$.

Figure S9-S10. ^{13}C NMR and IR spectra of **1**.

Figures S11-S13. ^{1}H, ^{13}C NMR and IR spectra of **2**.

Figures S14-S16. ^{1}H, ^{13}C NMR and IR spectra of **3**.

II. Crystallographic Details

Table S1 Crystal data for compound **2** and **3**

III. Polymerization Studies

Figure S17. MWDs of cPLA obtained by reaction of *L*-LA and **2** and **3**.

Figure S18. MWDs of cPLA obtained by reaction of *L*-LA and **2** (200:1+200+200).

Figure S19. ^{1}H NMR (300 MHz, C_6D_6, 300 K) spectrum of cPLA obtained by reaction of *L*-LA and **2** in ratio [monomer]:[Zn] = 200:1 at 100 °C in toluene.

Figure S20. MALDI-ToF spectrum of cyclic-PLLA obtained by reaction of *L*-LA and **2** in ratio [monomer]:[Zn] = 50:1 at 100 °C in toluene.

Figure S21. IR spectrum of cyclic-PLLA obtained by reaction of *L*-LA and **2** in ratio [monomer]:[Zn] = 50:1 at 100 °C in toluene.

Figure S22. MALDI-ToF spectrum of linear PLLA obtained by reaction of *L*-LA and **3** in ratio [monomer]:[Zn] = 50:1 at 100 °C in toluene.

Figure S23. IR spectrum of linear PLLA obtained by reaction of *L*-LA and **3** in ratio [monomer]:[Zn] = 50:1 at 100 °C in toluene.

I. Spectroscopic Characterization

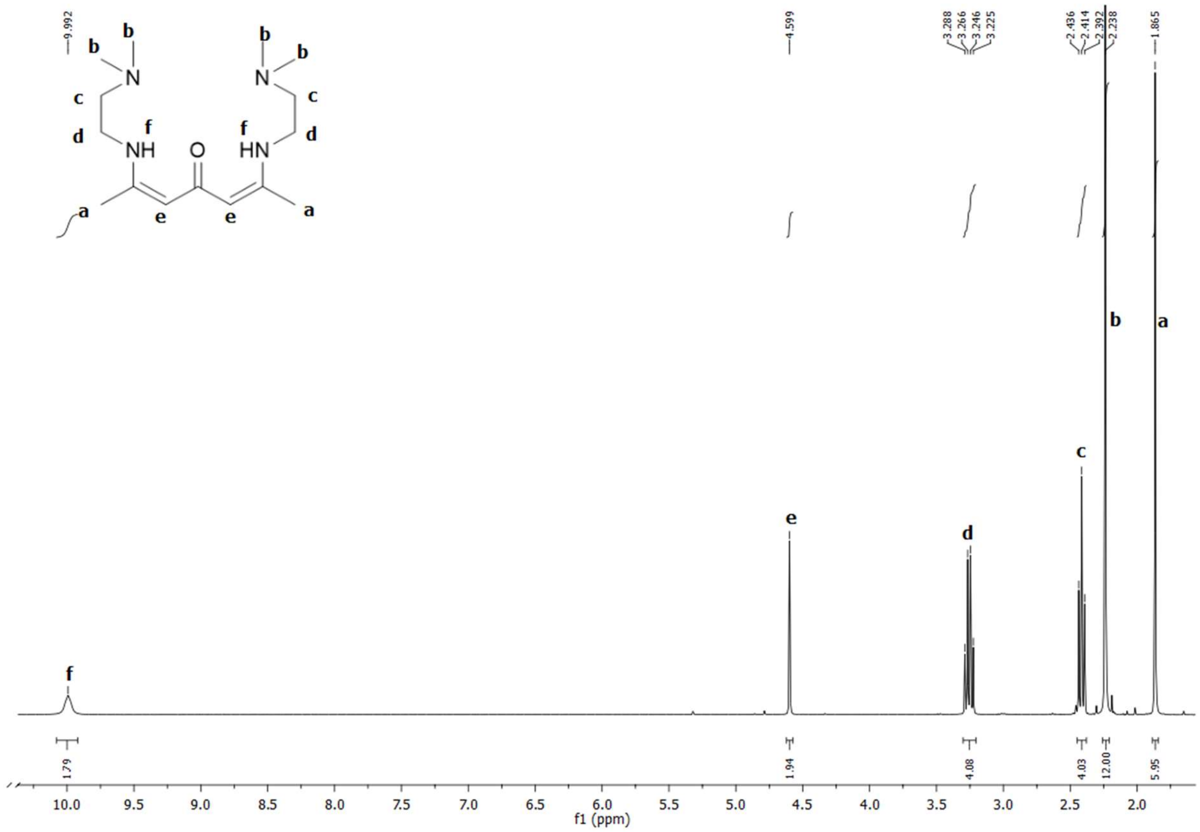

Figure S1. ^{1}H NMR (300 MHz, CD_2Cl_2, 25 °C) spectrum of L^1H_2.

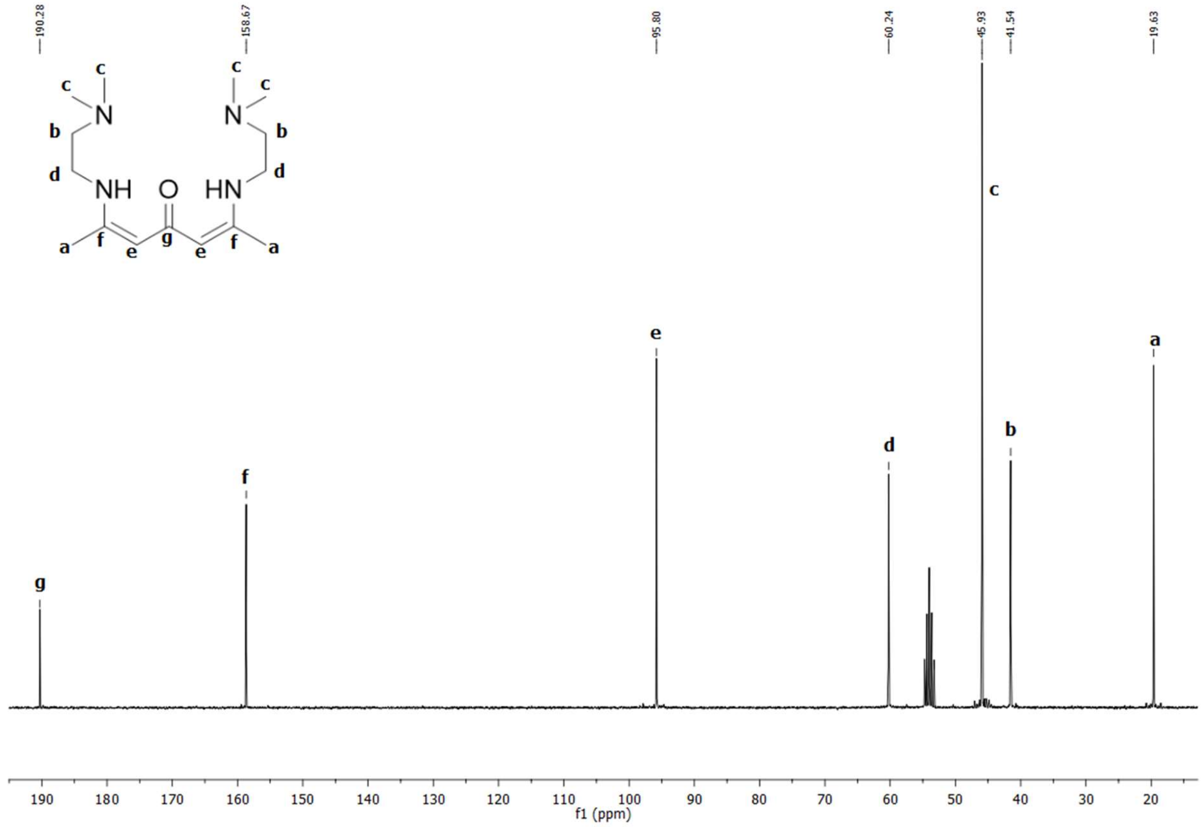

Figure S2. ^{13}C NMR (75 MHz, CD_2Cl_2, 25 °C) spectrum of L^1H_2.

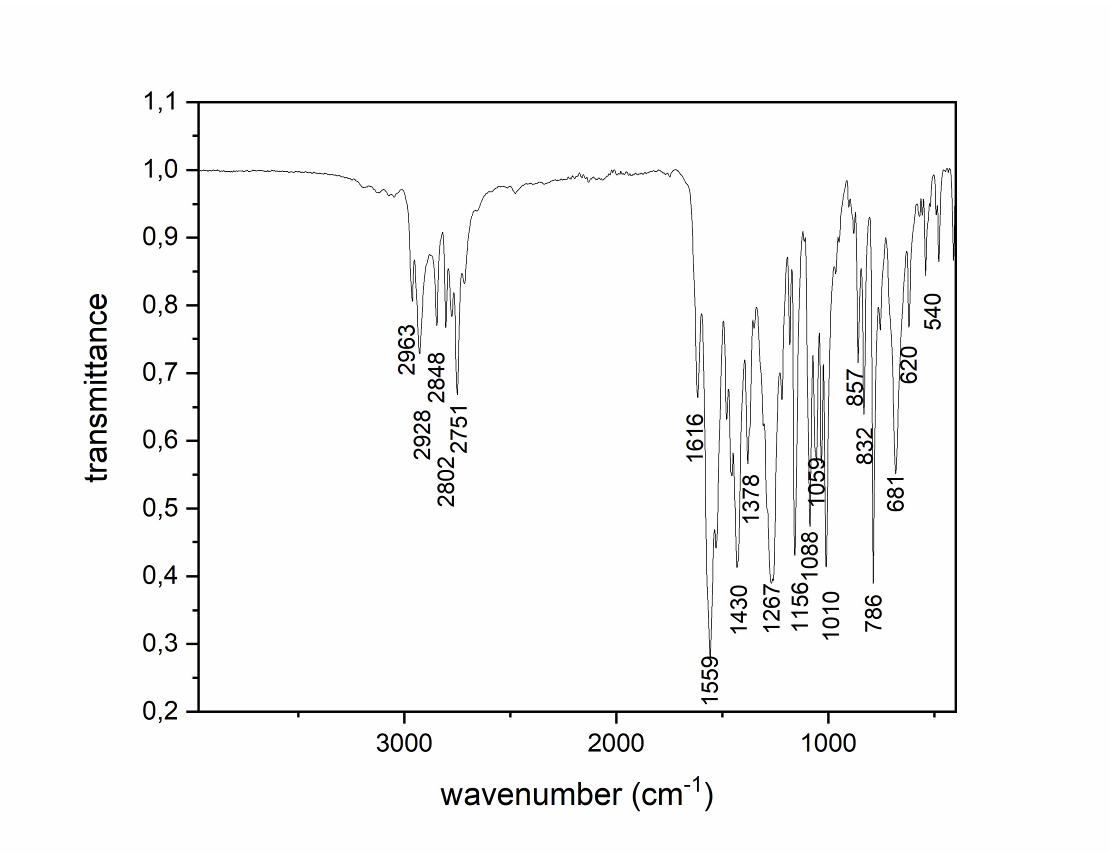

Figure S3. IR spectrum of L^1H_2.

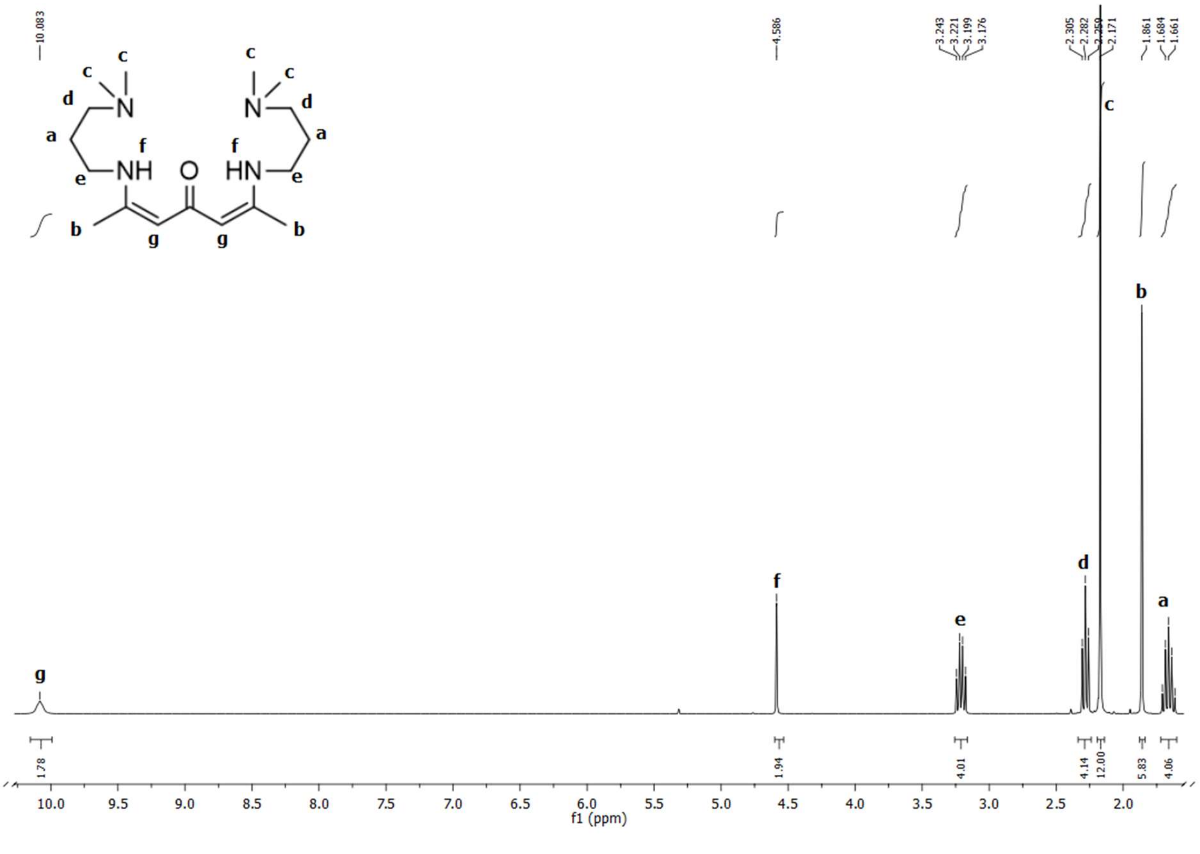

Figure S4. ^{1}H NMR (300 MHz, CD_2Cl_2, 25 °C) spectrum of L^2H_2.

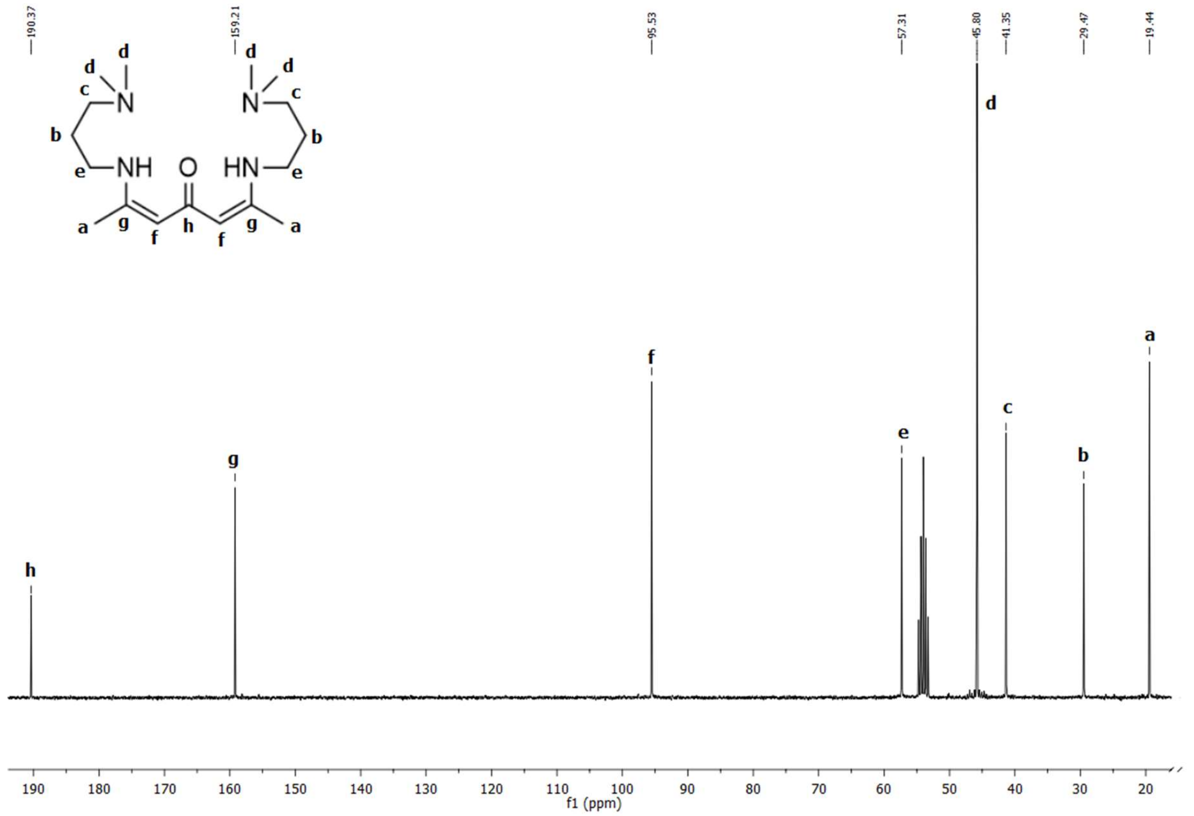

Figure S5. ^{13}C NMR (75 MHz, CD_2Cl_2, 25 °C) spectrum of L^2H_2.

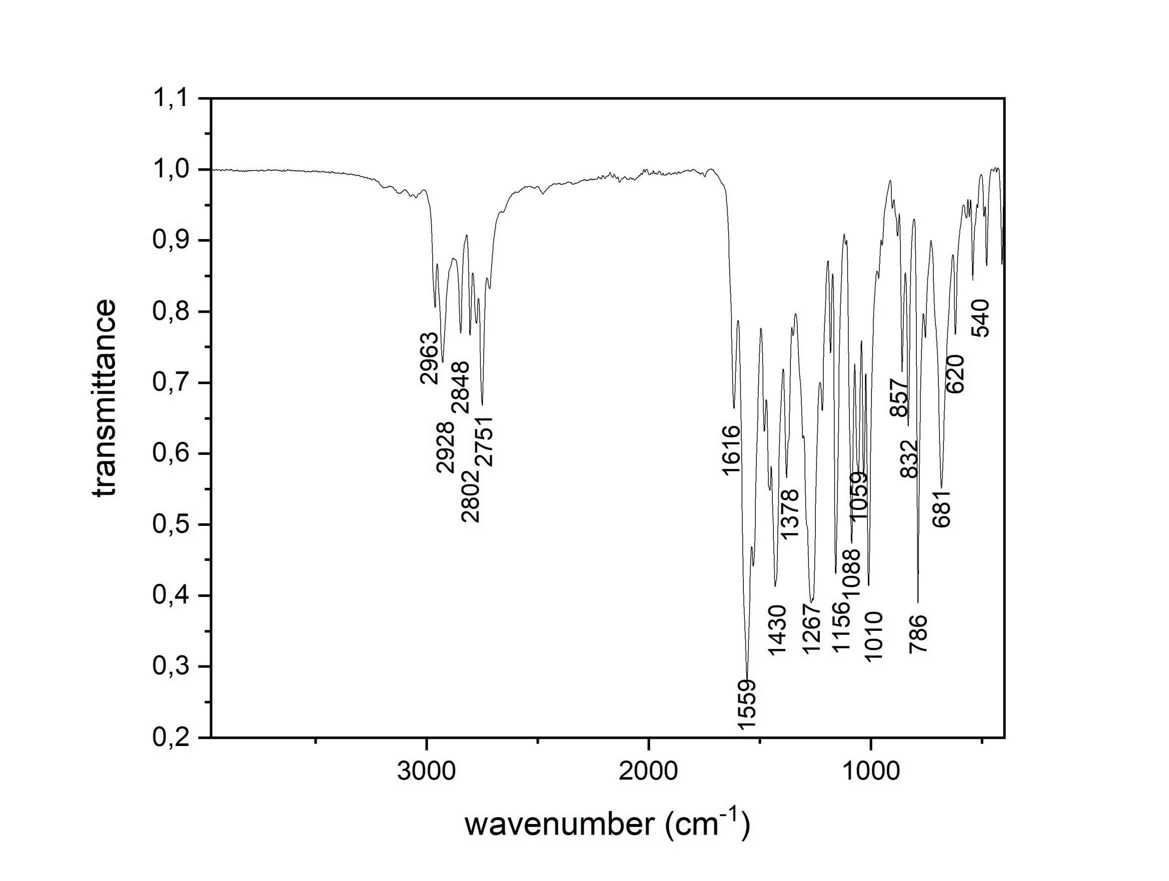

Figure S6. IR spectrum of L^1H_2.

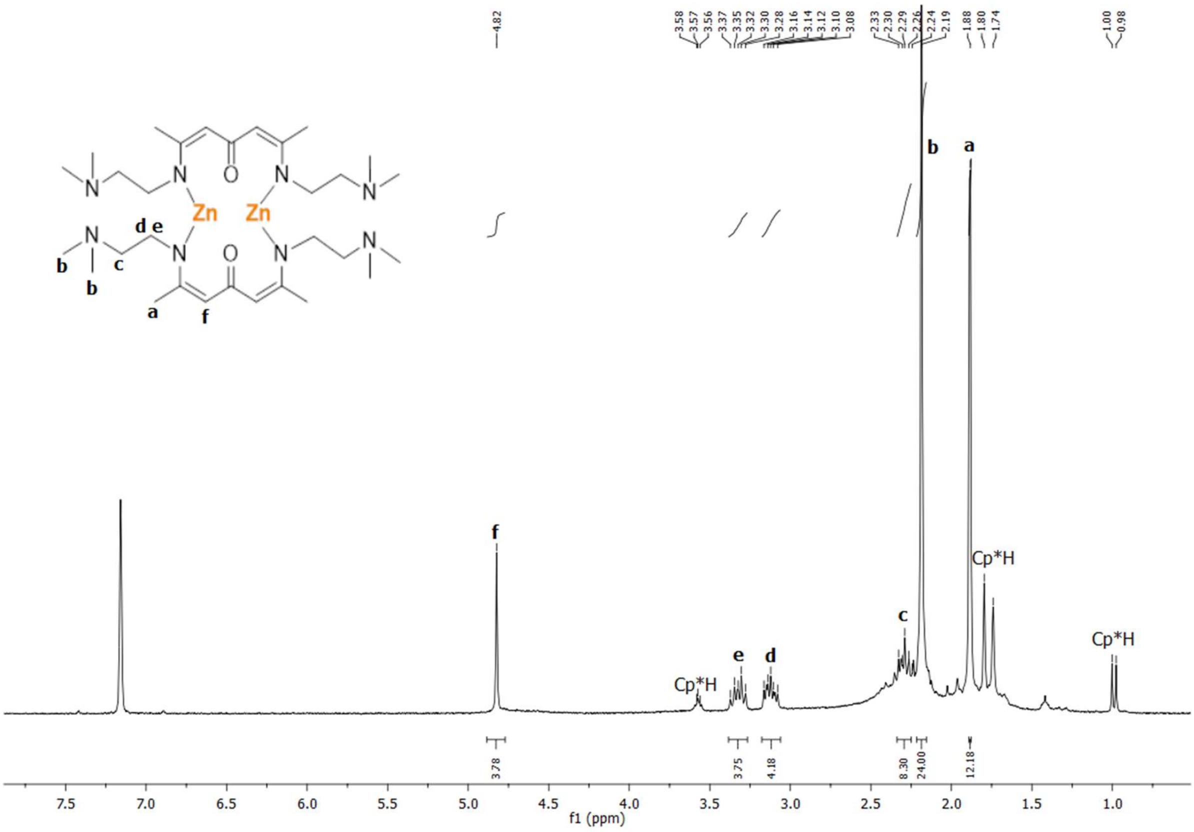

Figure S7. ^{1}H NMR (300 MHz, CD_2Cl_2, 25 °C) spectrum of the reaction of L^1H_2 with 2 eq. of $ZnCp^*_2$ yielding compound **1** and Cp*H.

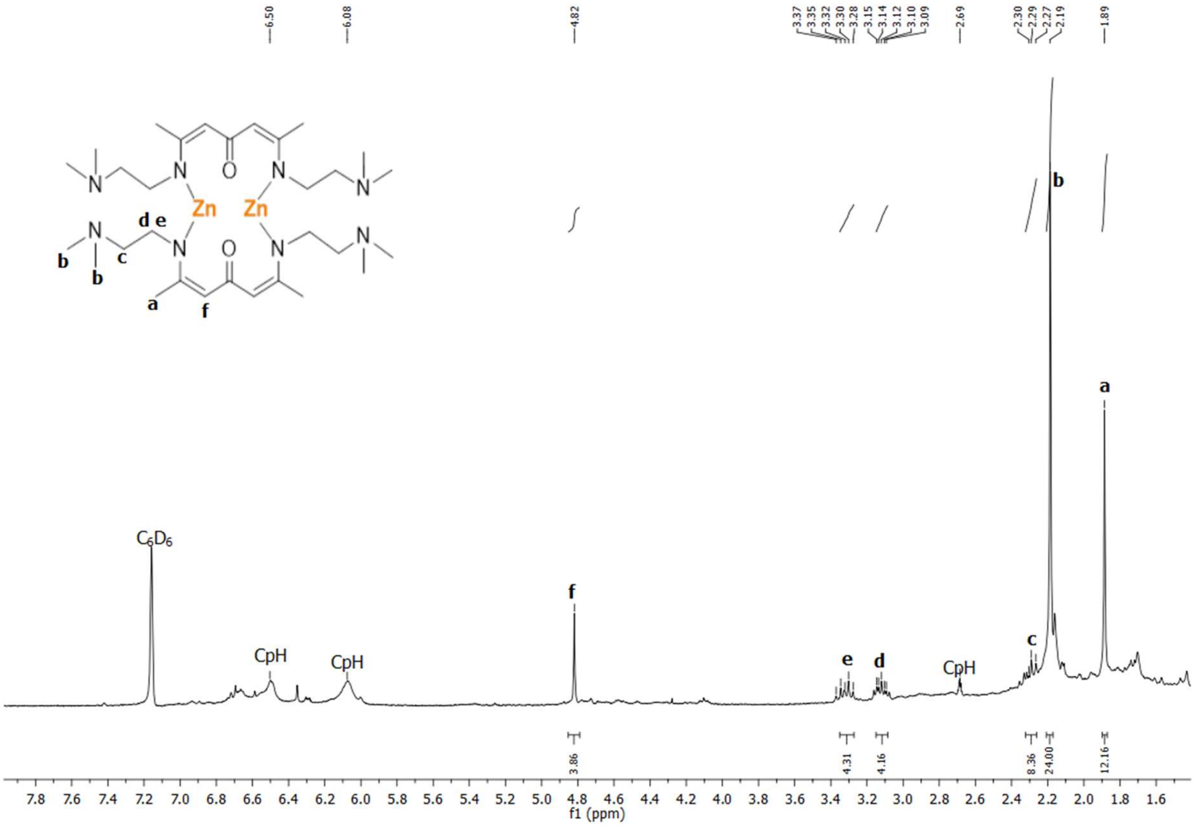

Figure S8. ^{1}H NMR (300 MHz, CD_2Cl_2, 25 °C) spectrum of the reaction of L^1H_2 with 2 eq. of $ZnCp_2$ yielding compound 1 and Cp*H.

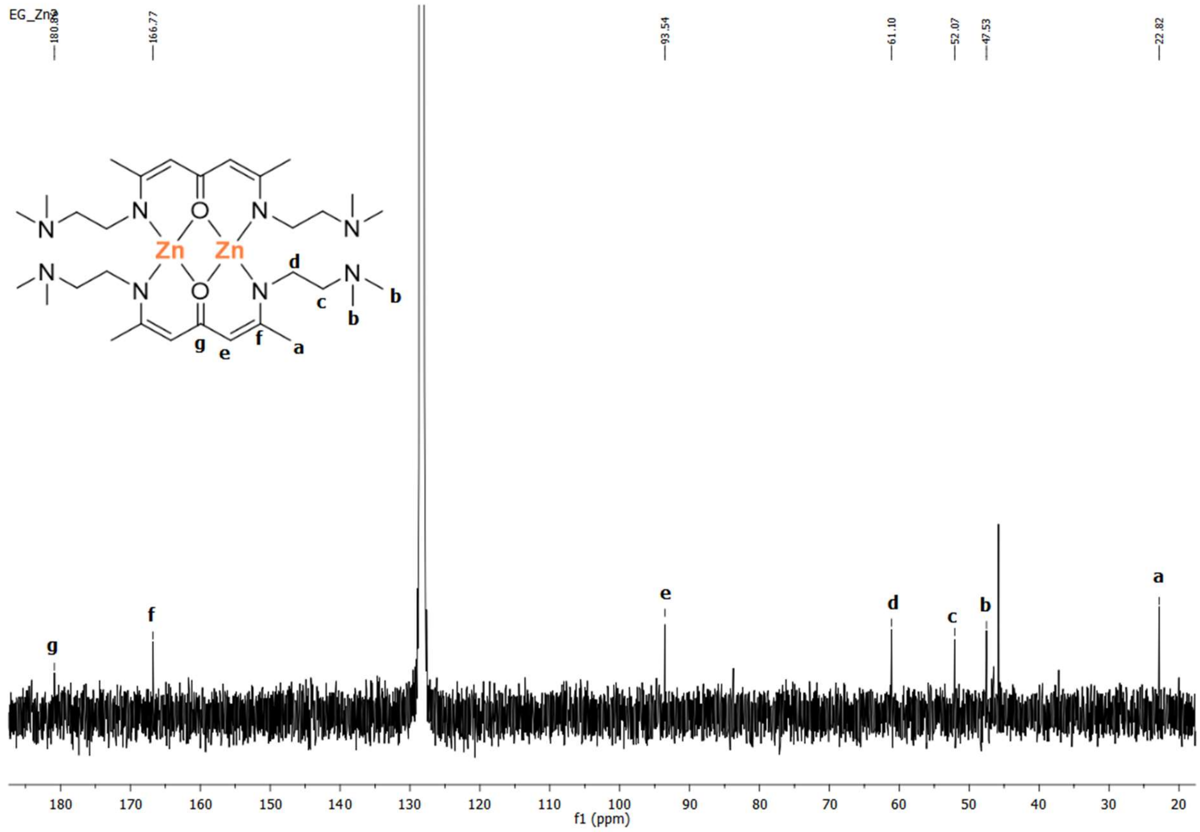

Figure S9. ^{13}C NMR (75 MHz, CD_2Cl_2, 25 °C) spectrum of **1**.

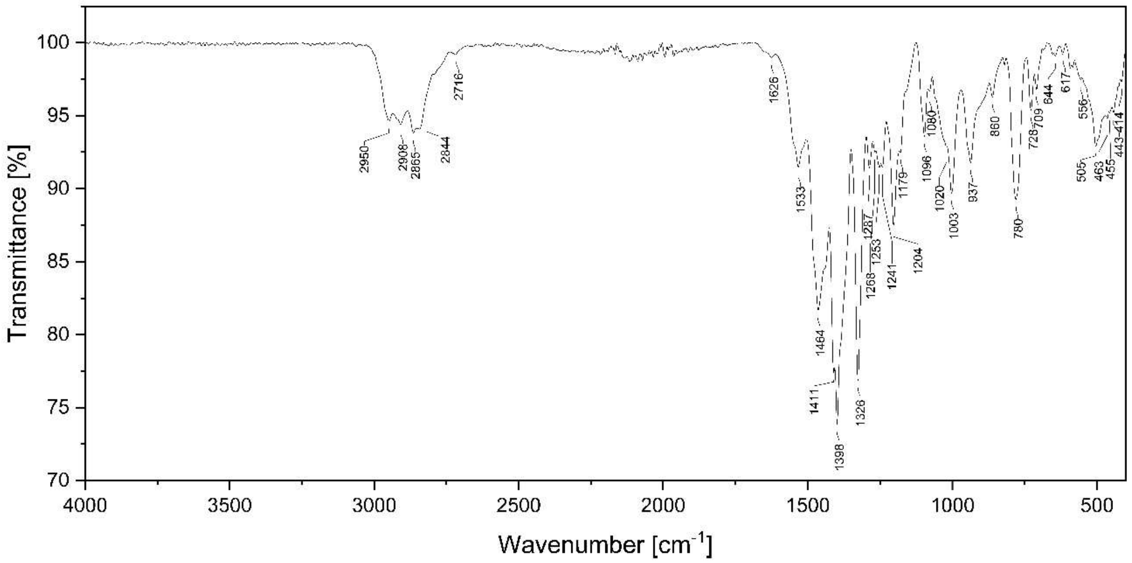

Figure S10. IR spectra of **1**.

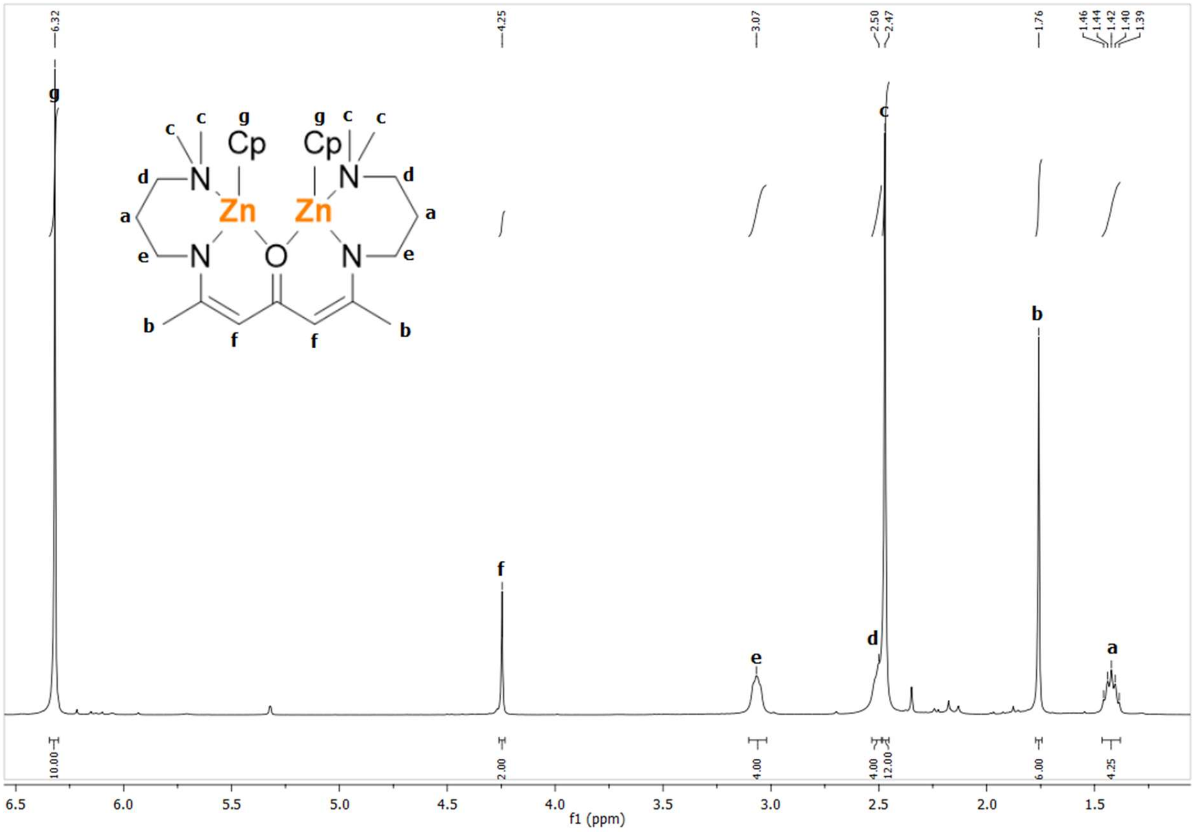

Figure S11. ^{1}H NMR (300 MHz, toluene-d_8, 25 °C) spectrum of **2**.

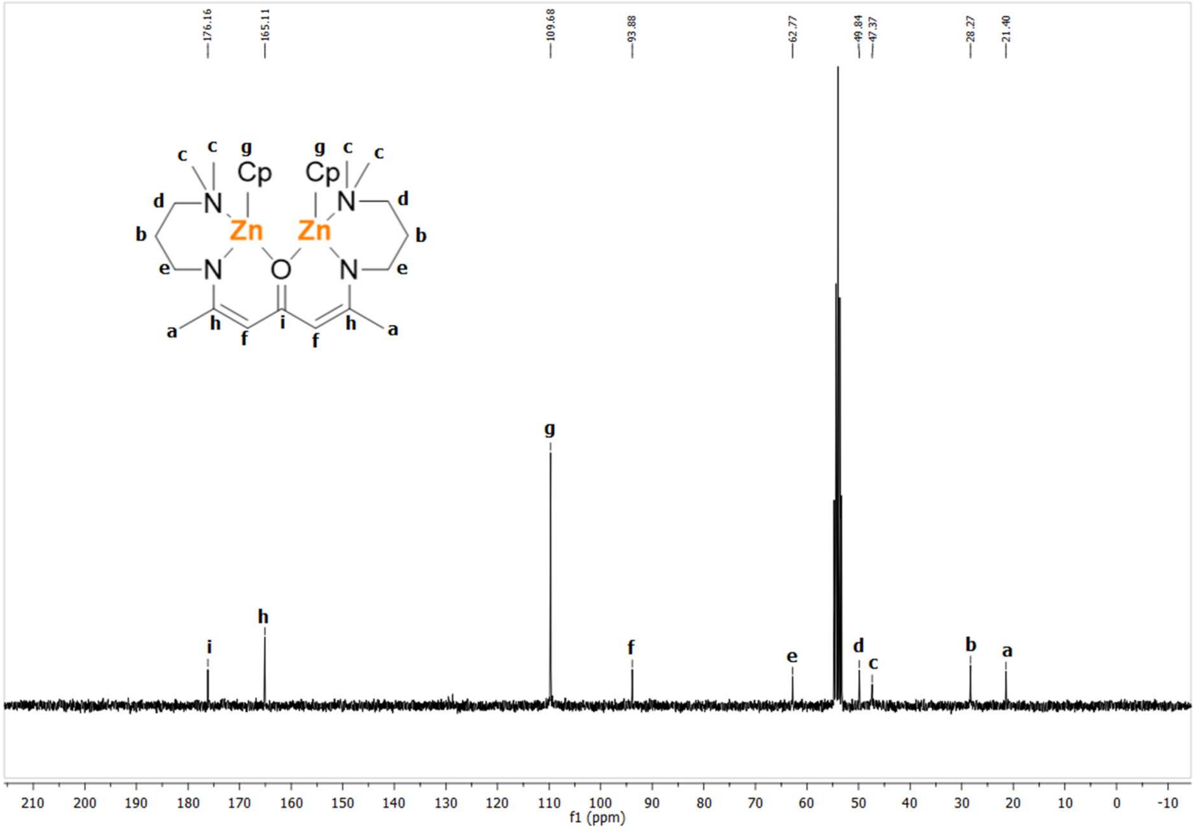

Figure S12. ^{13}C NMR (75 MHz, toluene-d_8, 25 °C) spectrum of **2**.

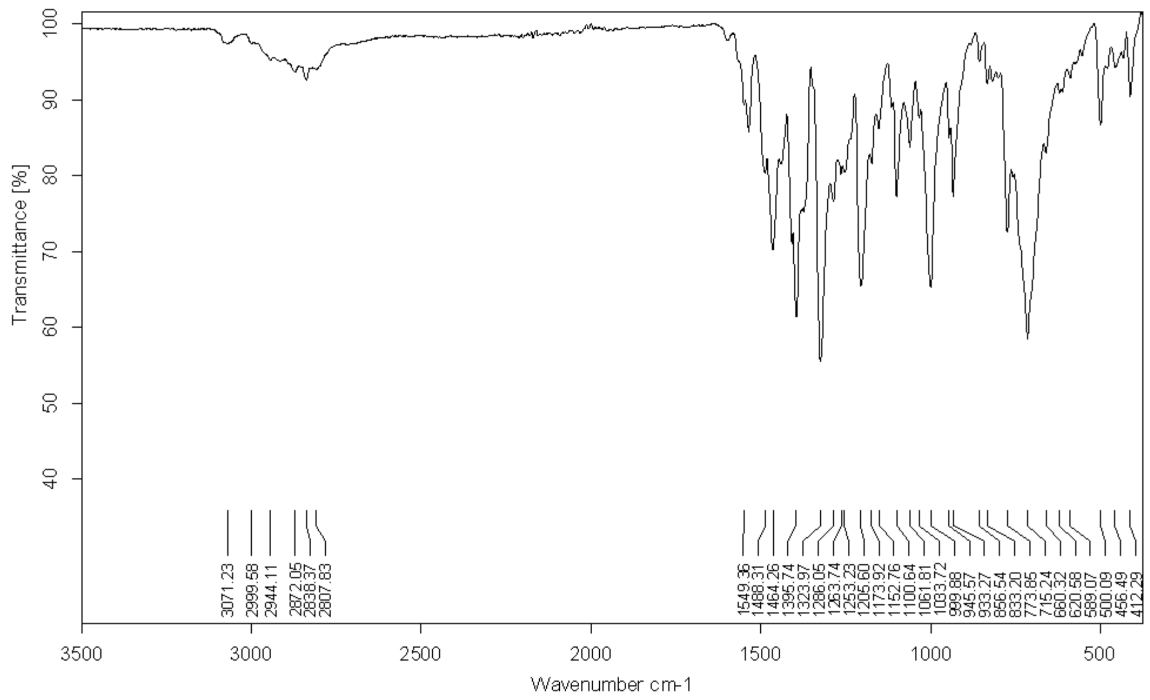

Figure S13. IR spectrum of **2**.

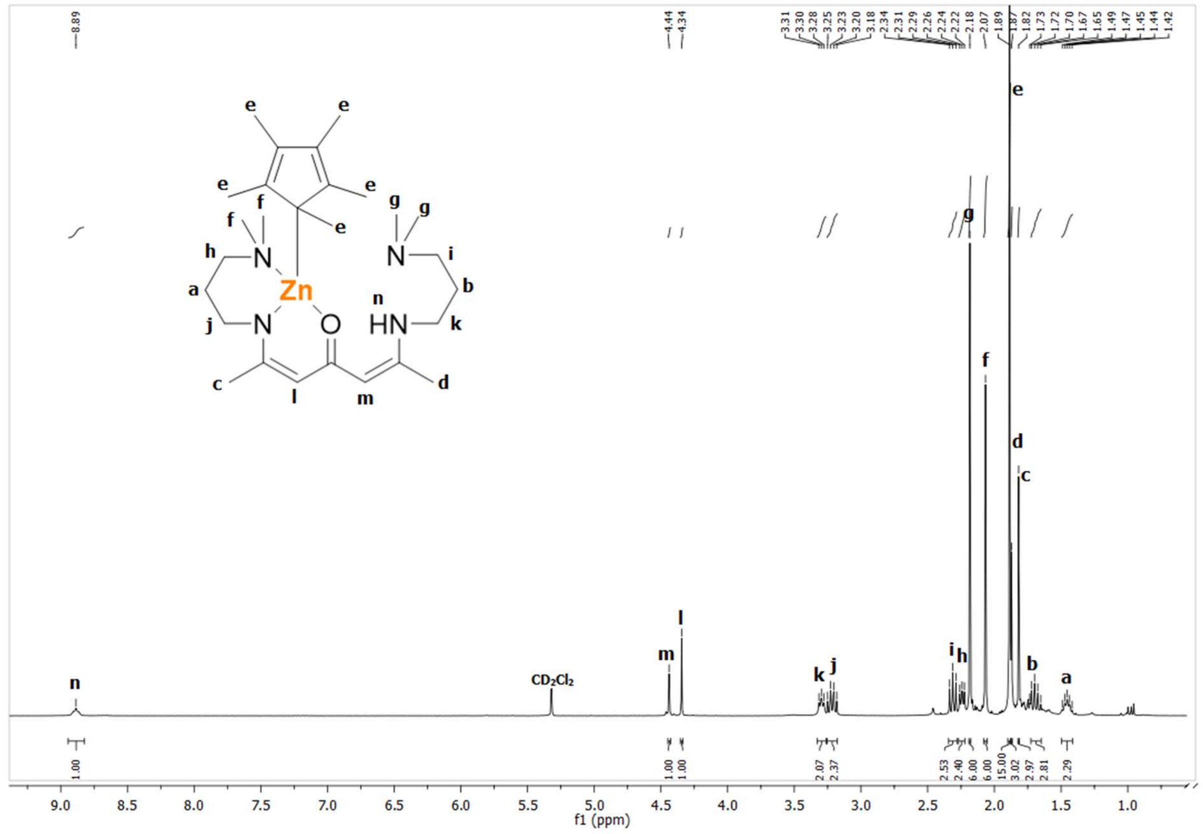

Figure S14. ^{1}H NMR (300 MHz, C_6D_6, 25 °C) spectrum of **3**.

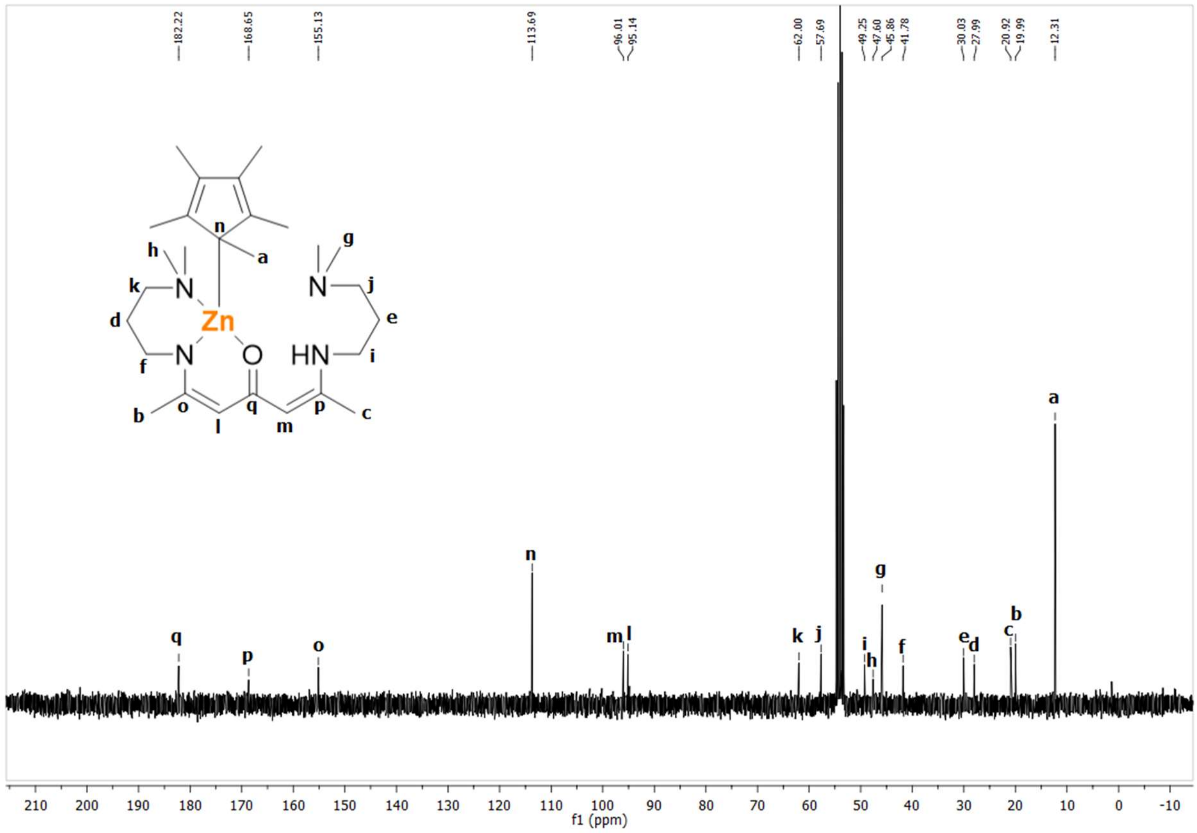

Figure S15. ^{13}C NMR (75 MHz, C_6D_6, 25 °C) spectrum of **3**.

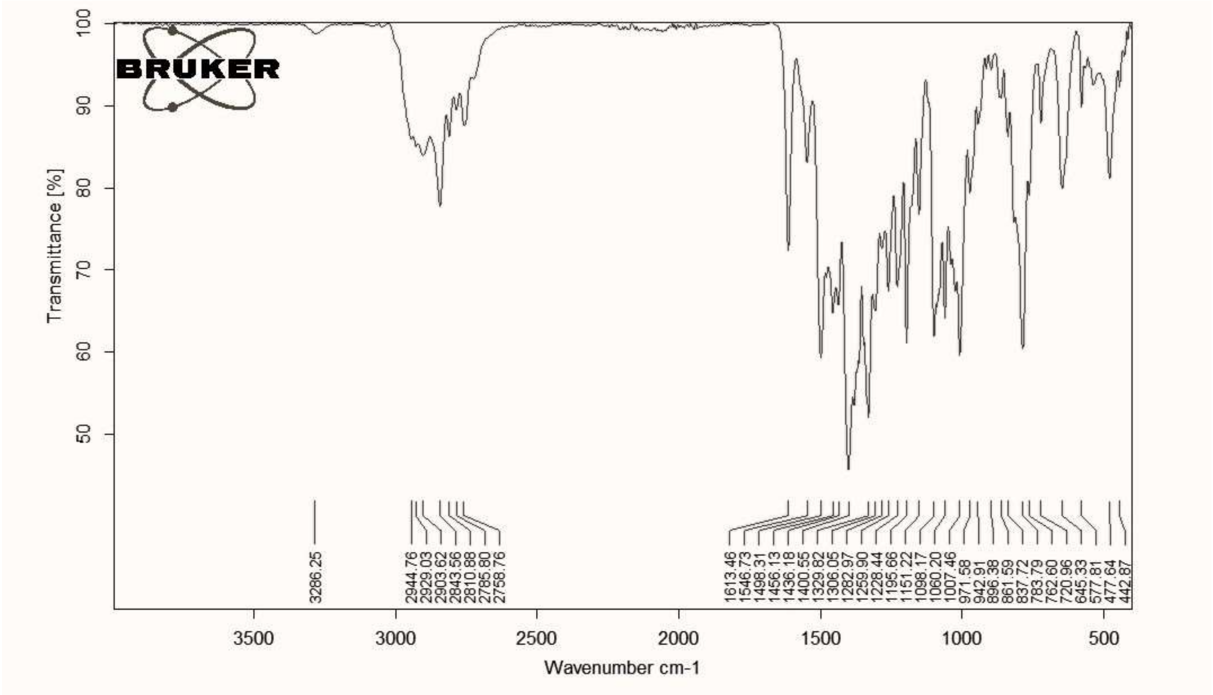

Figure S16. IR spectrum of **3**.

II. Crystallographic Details

Table S1 Crystal data for compounds **2** and **3**

Compound	**2**	**3**
Empirical formula	$C_{27}H_{42}N_4OZn_2$	$C_{27}H_{48}N_4OZn$
Formula weight (Da)	569.38	510.06
T (K)	100(2)	100(2)
Wavelength (Å)	0.71073	1.54178
Crystal system	triclinic	triclinic
Space group	*P*-1	*P*-1
a /Å	7.9884(6)	8.0513(4)
b /Å	8.0941(7)	12.3749(6)
c /Å	22.3164(18)	14.7581(7)
α (°)	98.388(4)	81.3299(17)
β (°)	95.725(4)	81.6550(17)
γ (°)	106.622(4)	74.9858(17)
V ($Å^3$)	1352.54(19)	1395.29(12)
Z, Calc. density (g cm^{-3})	2, 1.398	2, 1.214
Abs. coefficient (mm^{-1})	1.799	1.397
Crystal size (mm)	0.255 × 0.119 × 0.05	0.263 × 0.087 × 0.075
Theta range for data collection (°)	2.664°-33.268°	3.048°-79.538°
Reflections collected	92625	71276
Independent reflections	10387	5871
Data/restraints/parameters	10387/0/313	5871/52/362
Goodness-of-fit on F^2	1.040	1.062
Final *R* indices [$I > 2\sigma(I)$]	*R*1 = 0.029	*R*1 = 0.038
	*wR*2 = 0.064	*wR*2 = 0.099
R indices (all data)	*R*1 = 0.0406	*R*1 = 0.0421
	*wR*2 = 0.0679	*wR*2 = 0.1040

III. Polymerization Studies

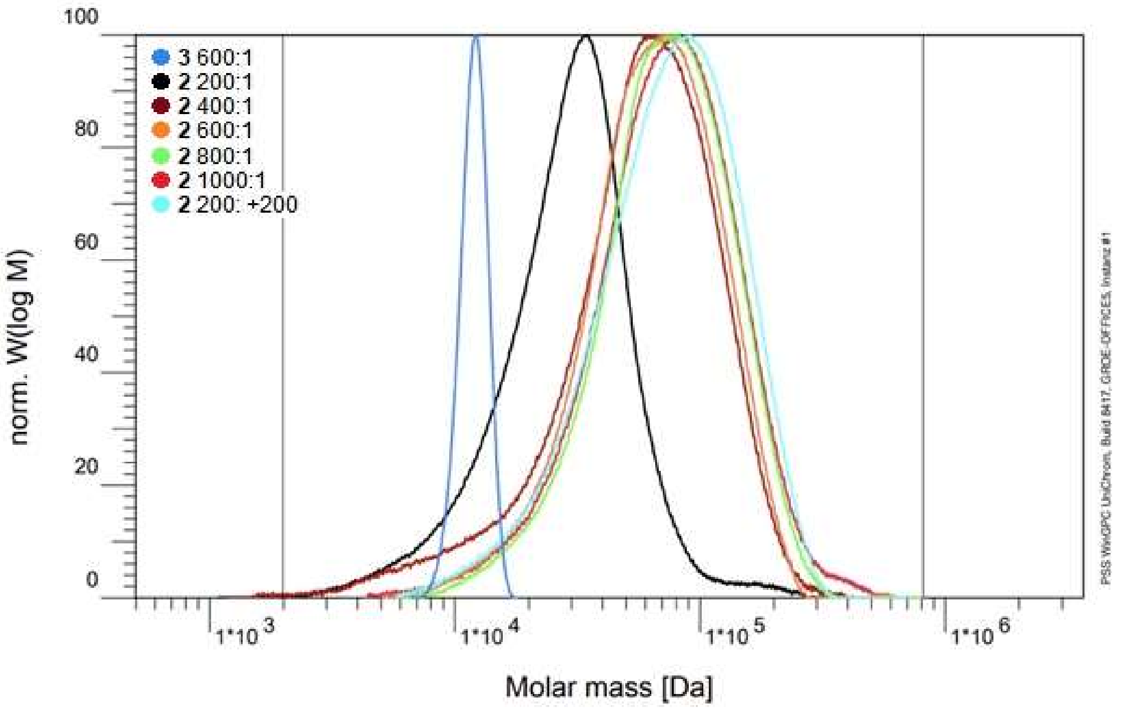

Figure S17. MWDs of cPLA obtained by reaction of *L*-LA and **2** and **3**.

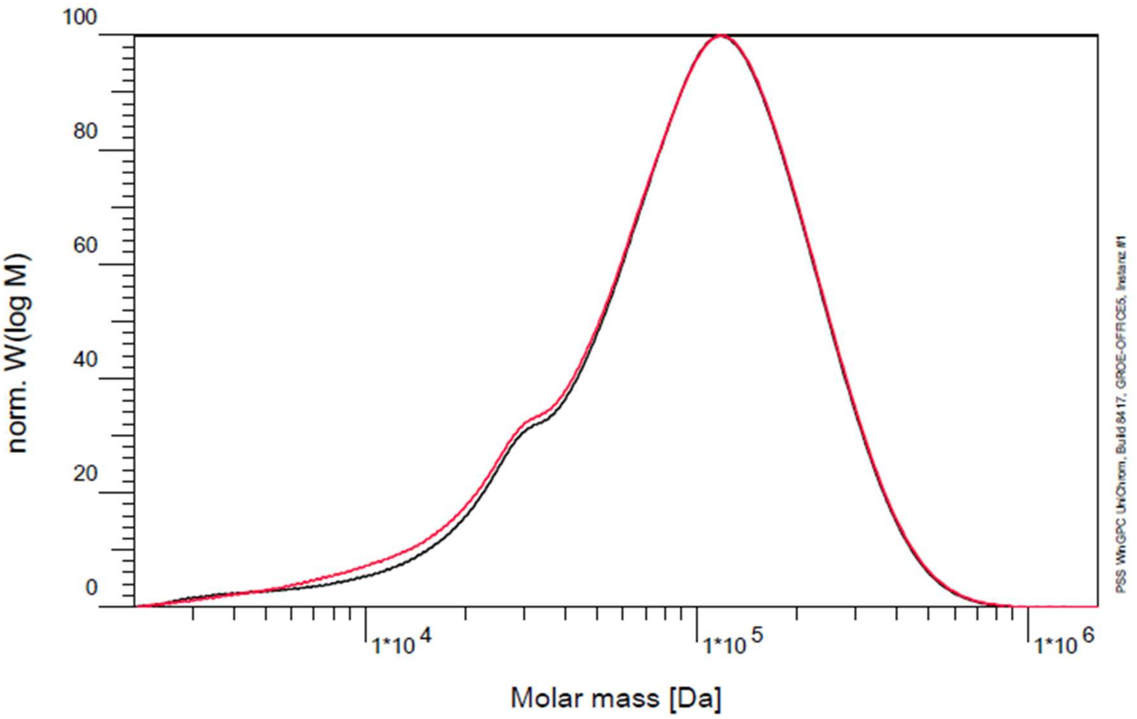

Figure S18. MWDs of cPLA obtained by reaction of *L*-LA and complex **2** (200:1+200+200).

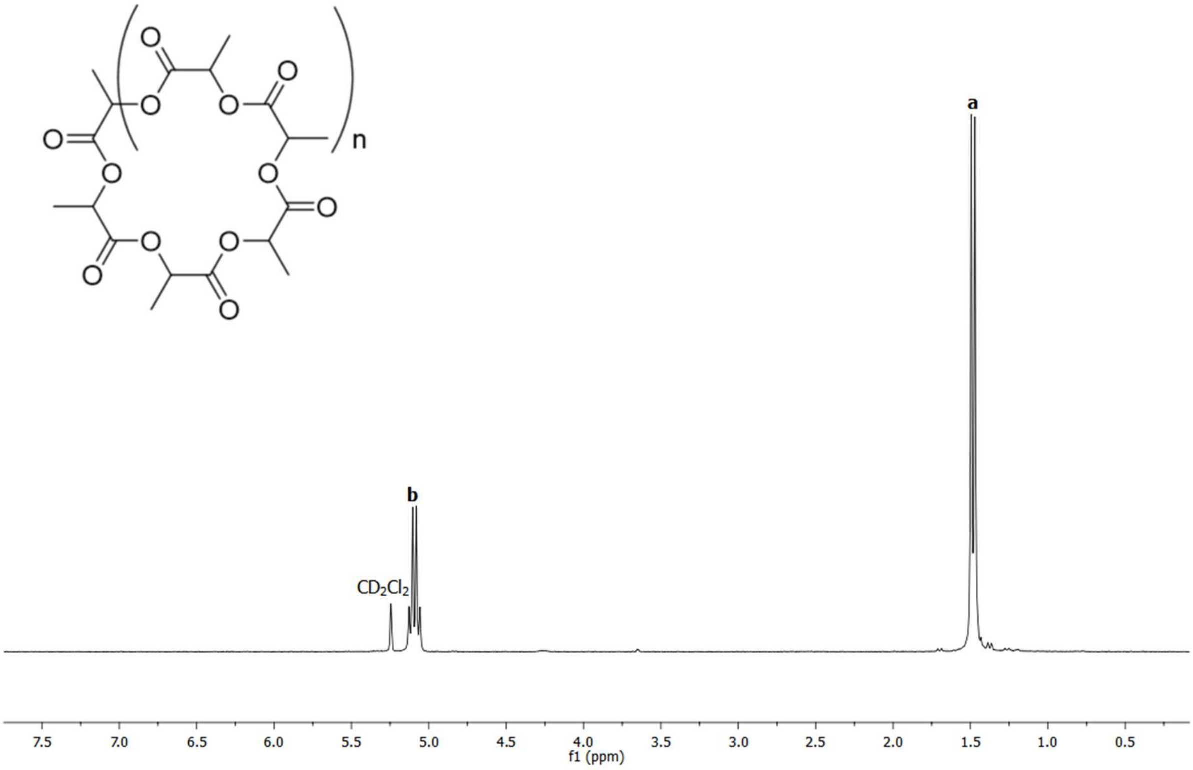

Figure S19. ^{1}H NMR (300 MHz, C_6D_6, 25 °C) spectrum of cPLA obtained by reaction of *L*-LA and **2** in ratio [monomer]:[Zn] = 200:1 at 100 °C in toluene.

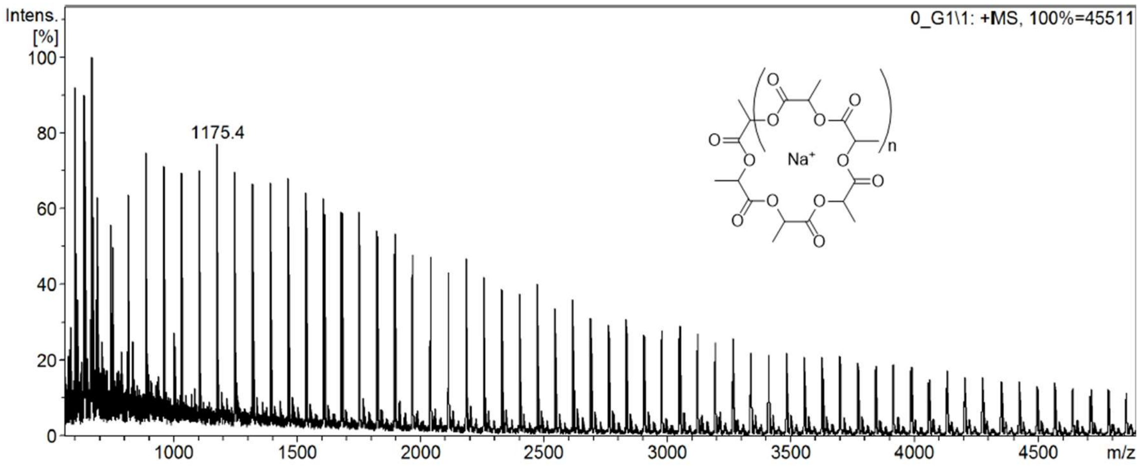

Figure S20. MALDI-ToF spectrum of cyclic-PLLA obtained by reaction of *L*-LA and **2** in ratio [monomer]:[Zn] = 50:1 at 100 °C in toluene.

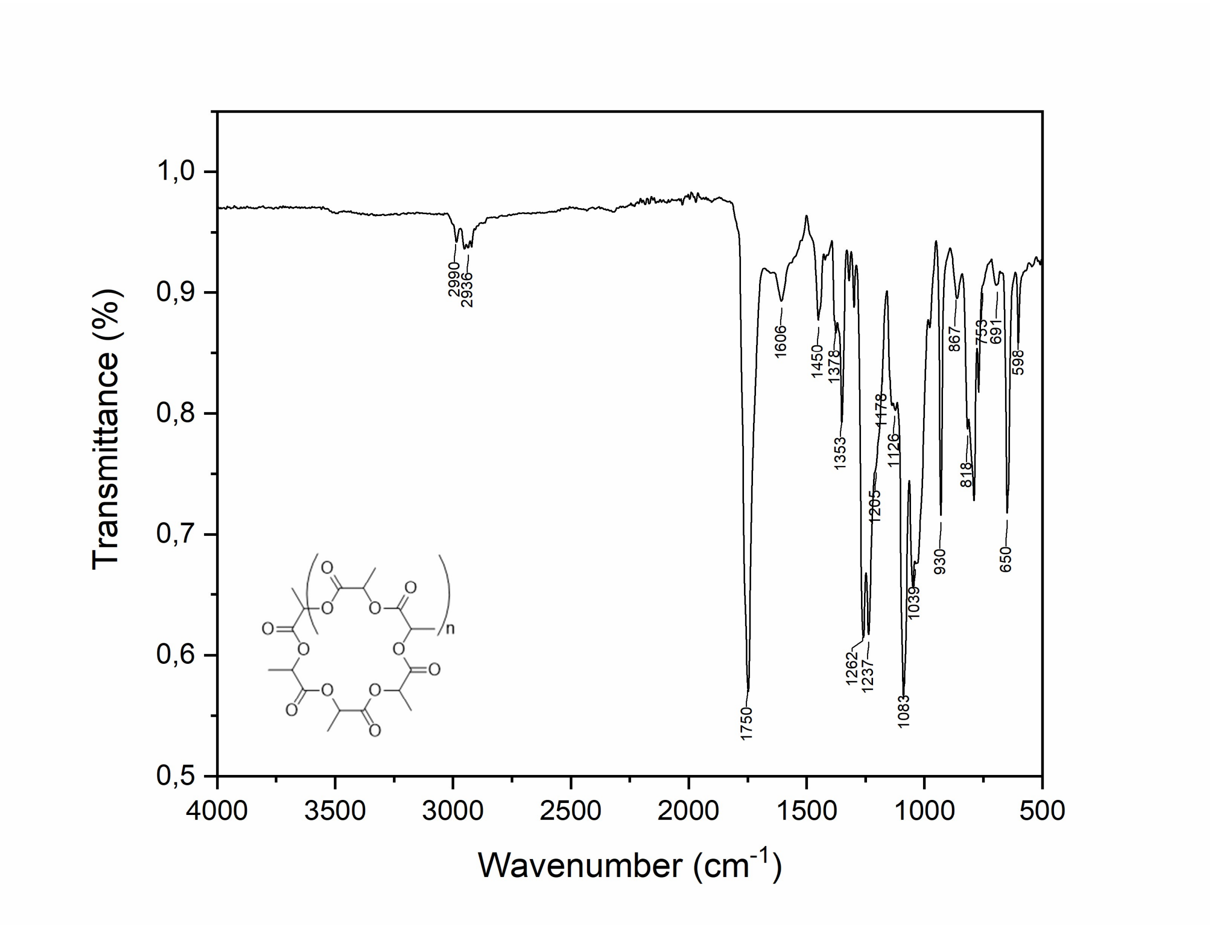

Figure S21. IR spectrum of cyclic-PLLA obtained by reaction of *L*-LA and **2** in ratio [monomer]:[Zn] = 50:1 at 100 °C in toluene.

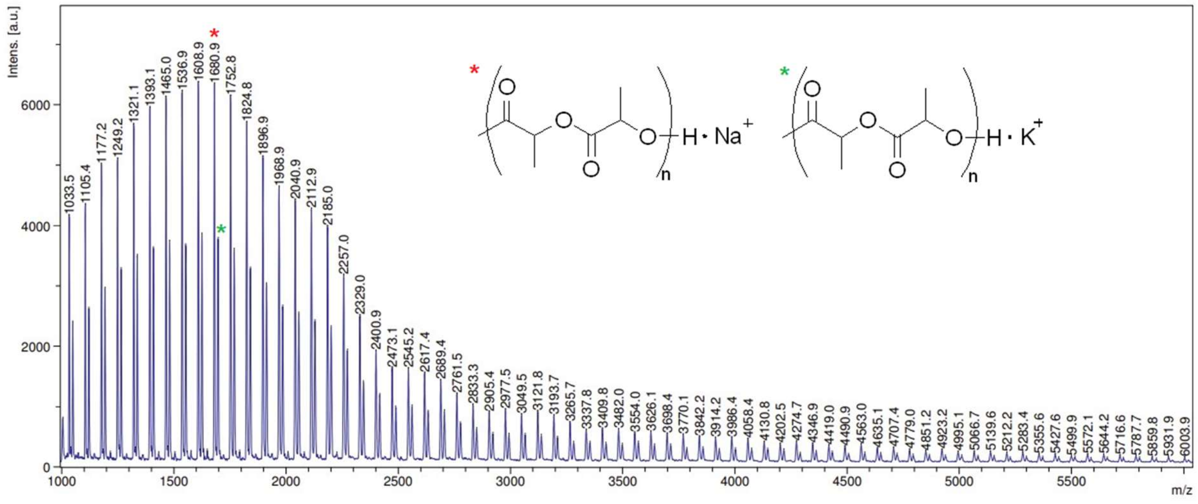

Figure S22. MALDI-ToF spectrum of linear PLLA obtained by reaction of *L*-LA and **3** in ratio [monomer]:[Zn] = 50:1 at 100 °C in toluene.

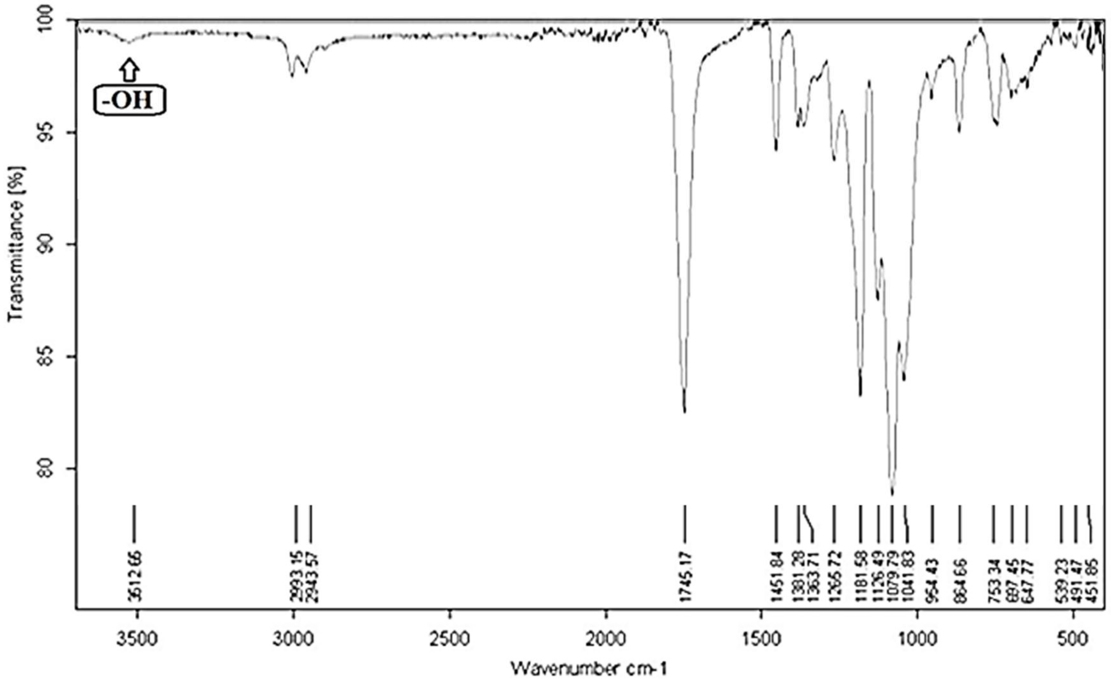

Figure S23. IR spectrum of linear PLLA obtained by reaction of *L*-LA and **3** in ratio [monomer]:[Zn] = 50:1 at 100 °C in toluene.
